# Hypobaric hypoxia-driven energy metabolism disturbance facilitates vascular endothelial dysfunction

**DOI:** 10.1016/j.redox.2025.103675

**Published:** 2025-05-17

**Authors:** Yuyu Zhang, Jinghuan Wang, Mengting He, Jiayao Liu, Jialin Zhao, JinTao He, Caiyun Wang, Yuhui Li, Chenxi Xiao, Chunxiang Fan, Jun Chang, Xinhua Liu

**Affiliations:** Phenome Research Center of TCM, Department of Traditional Chinese Medicine, Shanghai Pudong Hospital, Pharmacophenomics Laboratory, Human Phenome Institute, Fudan University, Shanghai, China

**Keywords:** High-altitude, Hypobaric hypoxia, Vascular endothelial dysfunction, Energy metabolism, PKM2, Lactate

## Abstract

Hypobaric hypoxia in plateau environments inevitably disrupts metabolic homeostasis and contributes to high-altitude diseases. Vascular endothelial cells play a crucial role in maintaining vascular homeostasis. However, it remains unclear whether hypoxia-mediated changes in energy metabolism compromise vascular system stability and function. Through integrated transcriptomic and targeted metabolomic analyses, we identified that hypoxia induces vascular endothelial dysfunction via energy metabolism dysregulation. Specifically, hypoxia drives a metabolic shift toward glycolysis over oxidative phosphorylation in vascular endothelial cells, resulting in excessive lactate production. This lactate overload triggers PKM2 lactylation, which stabilizes PKM2 by inhibiting ubiquitination, forming a feedforward loop that exacerbates mitochondrial collapse and vascular endothelial dysfunction. Importantly, blocking the pyruvate-lactate axis helps maintain the balance between glycolysis and oxidative phosphorylation, thereby protecting vascular endothelial function under hypoxic conditions. Our findings not only elucidate a novel mechanism underlying hypoxia-induced vascular damage but also highlight the pyruvate-lactate axis as a potential therapeutic target for preventing vascular diseases in both altitude-related and pathological hypoxia.

## Highlights

• Hypobaric hypoxia impairs vascular endothelium-dependent relaxation in mice.
• Hypoxia-mediated vascular endothelial dysfunction is associated with energy metabolism disorder.
• Hypoxia-promoted lactylation of PKM2 aggravates vascular endothelial dysfunction.
• Repair of the pyruvate-lactate axis can alleviate hypoxia-mediated vascular endothelial dysfunction.

## Graphical abstract

**Figure:**
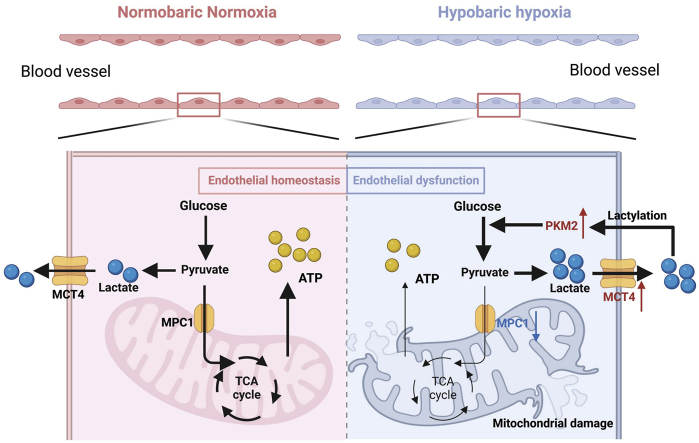

## 1 Introduction

The hypobaric hypoxia environment at high altitude can destroy vascular homeostasis, leading to cardiovascular complications of altitude diseases such as cerebral edema and pulmonary edema [1]. Vascular endothelial cells (VECs) are an important component in maintaining vascular homeostasis. Endothelial dysfunction is initially defined as the phenotype of decreased vasodilation along with cardiovascular diseases (CVDs) [2,3]. To some extent, endothelial dysfunction is regarded as a precursor of vascular disease. Hypoxia-inducing factor 1α (HIF-1α) stimulates angiogenesis by regulating vascular endothelial growth factor A, ANGPT2, and erythropoietin [4,5]. On the other hand, hypoxia disrupts vascular homeostasis by increased blood-brain barrier (BBB) permeability [6] and decreased vasodilation function. VECs play an important role in the local regulation of vascular tone, which is mainly dependent on endothelium-dependent nitric oxide synthase (eNOS). The hypobaric hypoxia environment in plateau not only affects the expression of eNOS, but also regulates the enzyme activity of eNOS through post-translational modifications [7]. These results indicate that hypoxia has a complex effect on VECs, but the specific mechanism is still unclear.

In high-altitude environment, hypoxia inevitably affects the energy metabolism of the body. Under normal conditions, glycolysis and oxidative phosphorylation are in a balanced state. When the body is in a state of hypoxia, the body's energy metabolism tends to glycolysis without consuming oxygen to meet the body's energy needs [8]. Glucose is first converted to pyruvate by glycolysis in the cytoplasm. This pyruvate is taken up by mitochondria via mitochondrial pyruvate carrier (MPC) [9] and subsequently enters the tricarboxylic acid cycle (TCA). Pyruvate can also convert lactate in the cytoplasm via lactate dehydrogenase (LDH), lactate then is transported out of the cell by monocarboxylate transporters (MCT) [10]. In fact, the homeostasis between pyruvate and lactate, referred to as the pyruvate-lactate axis, plays a significant role in cardiac function [11]. Hypoxia disrupts the balance between glycolysis and oxidative phosphorylation. Studies have shown that HIF-1α regulates cellular energy metabolism by inducing genes associated with glycolysis pathways such as phosphor-fructose kinase (PFK), lactate dehydrogenase (LDHA) and MCT4 [12]. Endothelial cells exhibit unique metabolic dependence, primarily relying on glycolysis for ATP production [13]. Although HIF drives adaptive metabolism toward glycolysis [8], sustained hypoxia disrupts the balance between glycolysis and oxidative phosphorylation, however the mechanisms linking these metabolic perturbations to vascular endothelial dysfunction remain unclear.

Both environmental hypoxia and pathological hypoxia disrupt vascular endothelial homeostasis, and the role of metabolic reprogramming in vascular endothelial dysfunction remains unclear. Elucidating the underlying mechanisms holds promise for identifying potential therapeutic targets in cardiovascular diseases. In the present study, we performed an integrated analysis using transcriptomics and targeted metabolomics, and defined hypobaric hypoxia exposure caused vascular endothelium dysfunction by disturbance of energy metabolism. Further analysis revealed that the regulation of the pyruvate-lactate axis can maintain the balance between glycolysis and oxidative phosphorylation to protect vascular endothelial function under hypoxic condition. Our study elucidates the pivotal role of hypoxia-induced metabolic dysregulation in driving vascular endothelial dysfunction, unveiling novel therapeutic avenues for combating cardiovascular and cerebrovascular pathologies associated with hypoxia.

## 2 Materials and methods

### 2.1 Cell culture and hypoxia treatment

Rat thoracic aorta endothelial cells (RAECs) were isolated and cultured from the thoracic aorta of adult SD rats. In brief, adult rats were sacrificed by neck amputation, sterilized by immersion in 75 % alcohol, and the thoracic aorta was carefully dissected in an ultra-clean table. After removing the connective tissue from the vessels, the thoracic aorta was cut into vascular segments of about 1 cm. One section of the vessel was carefully ligated with a surgical thread to turn the endothelium upside down, and then the other section was also ligated. The vessel segments were incubated in ECM with 10 % fetal bovine serum (FBS) and antibiotic solution (100 U/mL penicillin, 0.1 mg/mL streptomycin) in a constant temperature incubator at 37 °C and 5 % CO_2_. When the RAECs crawl out and reached 90 % confluence, cells were subcultured after treatment with 0.25 % trypsin-EDTA mixture.

Human umbilical vein endothelial cells (HUVECs) were purchased from ATCC. HUVECs were incubated in ECM (Allcells) with 2 % fetal bovine serum (FBS) and antibiotic solution (100 U/mL penicillin, 0.1 mg/mL streptomycin) in a constant temperature incubator at 37 °C and 5 % CO_2_. When the HUVECs crawl out and reached 90 % confluence, cells were subcultured after treatment with 0.25 % trypsin-EDTA mixture.

When the degree of RAECs or HUVECs fusion reached about 80 %, the serum-free medium was replaced and incubated under 5 % O_2_ for 72 h.

### 2.2 Animal studies and drug treatment

All animals in the experiment were male C57BL/6J mouse aged 6–8 weeks. All animals and the experimental protocol conformed to the Animal Welfare Act Guide for Use and Care of Laboratory Animals, and were approved by Institutional Animal Care and Use Committee (IACUC), Fudan University, China.

The mice in the hypobaric hypoxia group were raised in the plateau simulated chamber (Guizhou Fenglei aviation ordnance Co., LTD) for 45 days, the altitude parameter was set to 6000 m, and the oxygen concentration was 9 %.

The Sodium dichloroacetate (DCA) was dissolved in ddH_2_O at a concentration of 0.5 mg/mL, and the administration time lasted throughout the hypobaric hypoxia treatment time for 45 days. UK-5099 (MedChemExpress, HY-15475) was dissolved in cosolvent according to instructions. The mice were given UK-5099 by intragastric administration for 7 and 14 days at a dose of 3 mg/kg/d. The control group received equal volumes of vehicle.

### 2.3 Small interfering RNA transfection in vitro

Mouse MCT4 (sense: CACGGCAGGUUUCAUAACATT, antisense: UGUUA UGAAACCUGCCGUGTT), MPC1 (sense: GCUAUUCUCUGACAUUCAUTT, antisense: AUGAAUGUCAGAGAAUAGCTT), PKM2 (sense: GCCACAGAAA GCUUUGCAUTT, antisense: AUGCAAAGCUUUCUGUGGCTT) and control small interfering RNA (siRNA) were obtained from GenePharma (Shanghai, China). When cells reached approximately 70 % confluence, complexes of Lipofectamine RNA iMAX (ThermoFisher Scientific, 13778075) and siRNA were added to the cells. After incubation under normal culture conditions for 24 h, the fresh medium was replaced, then the knockdown efficiency was assessed and follow-up experiments were performed.

### 2.4 Endothelium-dependent vasodilation test

The endothelium-dependent vasodilation function was studied in the isolated thoracic aorta of mice. In short, after killing the mice, the aorta was immediately removed and placed in a Krebs-Henseleit physiological salt solution of the following components (mmol/L): NaCl, 129; NaHCO_3_, 25; Glucose, 5.5; Potassium chloride, 4.7; Calcium chloride, 2.5; KH_2_PO_4_, 1.2; MgSO_4_, 1.17; EDTA, 0.025. The pH is 7.4 at 37 °C. The aorta was separated and the adjacent connective tissue was removed, then cut into 2 mm segments and fixed in the DMT 620 M angiotonometry system (Danish Myo Technology A/S Inc.) using 40 μm diameter stainless steel wire and the contraction forces were recorded. Each sample was balanced in Krebs solution for 60 min at 37 °C during which time it was aerated with a carbon gas mixture (5 % CO_2_ and 95 % O_2_) to maintain vascular activity. The initial vascular tension was set to 3 mN, then the value was reduced to zero, and the blood vessels were stimulated with a final concentration of 0.06 mM potassium chloride to test the vascular activity. This was followed by three washes with a buffer to fully dilate the blood vessels to baseline. Phenylephrine is added to stimulate vasoconstriction until equilibrium. Graded concentrations of acetylcholine were added successively to stimulate vasodilation and the data were recorded. The endothelium-dependent vasodilation was expressed as the percentage of preexisting contractile tension induced by Phenylephrine.

### 2.5 Measurement of mitochondrial membrane potential

Mitochondrial membrane potential was determined using the JC-10 Mitochondrial Membrane Potential Assay Kit (Yeasen, 40707ES08, China) according to manufacturer's instructions. In simple terms, the cells were washed with PBS and incubated with 10 μM JC-10 in the dark for 30 min. The fluorescence intensity of aggregate and monomer form JC-10 was determined by fluorescence microscopy (Carl Zeiss, Zeiss LSM780). ImageJ was used to quantify the red-green fluorescence ratio of some cells in a random field of view to characterize mitochondrial membrane potential.

### 2.6 Others

We provide detailed descriptions of the other methods and materials used in this article in supplemental materials.

### 2.7 Statistical analysis

Data are expressed as Mean ± SD. Unpaired *t*-test was used for two groups of data, and one-way ANOVA and Tukey-Kramer post hoc test were used for three or more groups of data. All analyses were performed using GraphPad Prism 8.0 statistical software package, and *p* < 0.05 was considered statistically significant.

## 3 Results

### 3.1 Combined transcriptomics and targeted metabolomics analysis reveals characterization of VECs upon hypoxia exposure

**Fig. 1:**
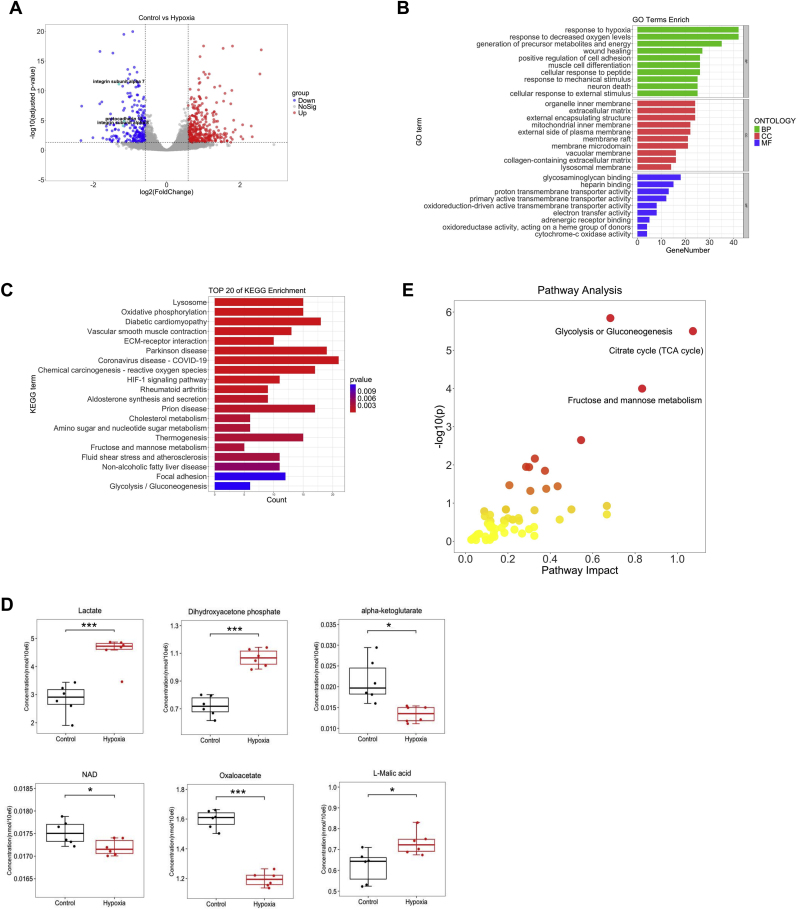
**Combined transcriptomics and targeted metabolomics analysis reveals characterization of vascular endothelial cells upon hypoxia exposure**. Rat aortic endothelial cells (RAECs) were cultured at 5 % O_2_ for 72 h and then subjected to RNA-seq analysis. Differentially expressed genes (DEGs) of RAECs (**A**); Gene ontology (GO) analysis of RAECs under hypoxic condition (**B**); KEGG functional enrichment analysis of RAECs (**C**). RAECs were cultured at 5 % O_2_ for 72 h and then subjected to targeted energy metabolomics analysis, differential metabolites detected in RAECs by targeted metabolomics. ∗*p* < 0.05, ∗∗∗*p* < 0.001 (**D**); MetPA analysis of RAECs under hypoxic condition (**E**).

To investigate the effects of hypoxia on VECs, we employed the RNA-seq technique to explore genetic differences between the control and hypoxia in RAECs. In order to identify differentially expressed genes (DEGs) related to endothelial function under hypoxic condition, we utilized DESeq2 to conduct RNA-seq dataset. Our analysis identified 610 DEGs with the fold change >1.5 and adjusted *p*-value <0.05 as cut-off, which comprising 380 up-regulated genes and 230 down-regulated genes (Fig. 1A, Supplemental Table 1). And the down-regulated genes included protocadherin, integrin subunit alpha 7 and integrin subunit alpha 8, which have been implicated in vascular impairment and endothelial barrier injury. Through Gene Ontology (GO) annotation, we observed that among the top 10 biological processes, the majority of annotated transcripts were associated with the hypoxia response. In terms of cellular components, the majority of annotated transcripts were found to correspond to the organ inner cortex, mitochondrial inner membrane and plasma membrane. Furthermore, molecular function analysis revealed that a significant number of annotated transcripts were involved in redox reactions (Fig. 1B). Kyoto Encyclopedia of Genes and Genomes (KEGG) functional enrichment analysis indicated that the DEGs were predominantly enriched in oxidative phosphorylation pathway, hypoxia-inducible factor-1 (HIF-1) pathway and focal adhesion pathway (Fig. 1C). In summary, transcriptomics analysis reveals that the hypoxia causes VECs function impairment characterization.

Most notably, the KEGG analysis of RNA-seq data involves oxidative phosphorylation pathway and glycolysis pathway. Therefore, we conducted targeted metabolomics of energy metabolism about samples of the control and hypoxic RAECs. This analysis successfully identified 23 metabolites with high confidence, including α-ketoglutarate, pyruvate, citrate and others (Fig. 1D–Supplemental Table 2). MetPA analysis demonstrated that these metabolites primarily participated in glycolysis, the TCA cycle, fructose and mannose metabolism pathways (Fig. 1E). This analysis suggests that the discrepancy characteristics of VECs caused by hypoxia are related to changes in energy metabolism.

### 3.2 Hypoxia leads to vascular endothelial dysfunction and energy metabolism disorders

**Fig. 2:**
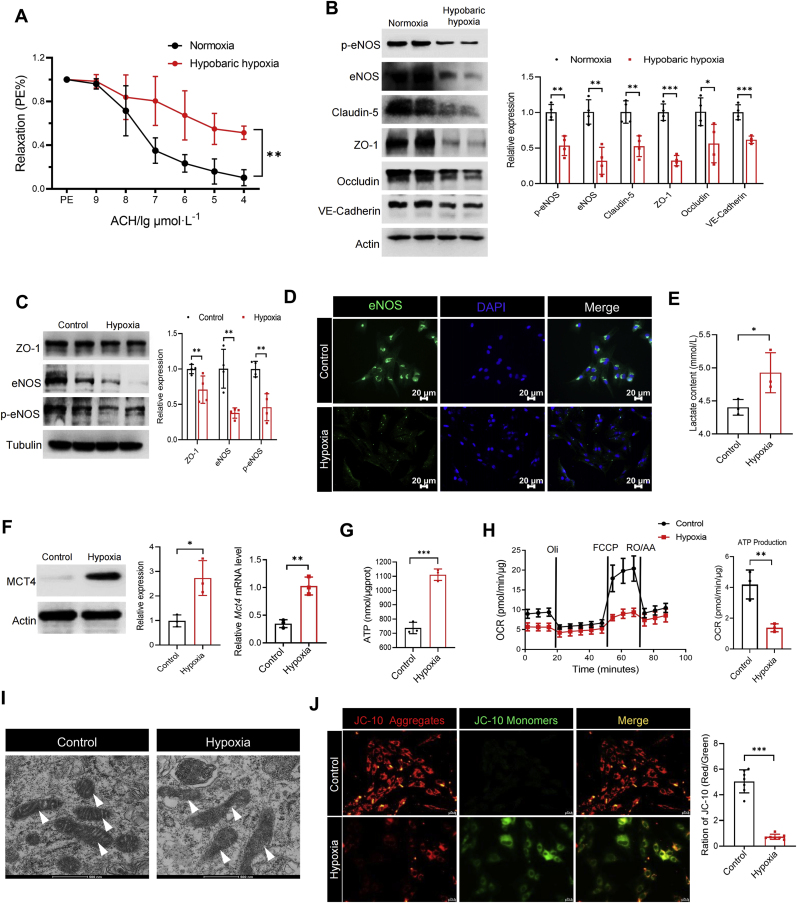
**Hypoxia leads to vascular endothelial dysfunction and energy metabolism disorders. (A**–**B)** Mice were placed for 45 days in a plateau simulated chamber with an oxygen concentration of about 9.0 % at an altitude of 6000 m. Endothelium-dependent relaxations to acetylcholine was measured using isolated aortic rings. ∗∗*p* < 0.01 (n = 6) (**A**); The protein expression of p-eNOS, eNOS, Claudin-5, ZO-1, Occludin and VE-Cadherin in thoracic aorta of mice were detected. ∗*p* < 0.05, ∗∗*p* < 0.01, ∗∗∗*p* < 0.001 (n = 4) **(B)**. **(C**–**J)** RAECs were cultured at 5 % O_2_ for 72 h. The protein expression of ZO-1, eNOS and p-eNOS was analyzed. ∗∗*p* < 0.01 (n = 4) (**C**); Representative images of immunofluorescence analysis of eNOS expression (**D**); Lactate content in the supernatant of RAECs culture. ∗*p* < 0.05 (n = 3) (**E**); Protein expression and mRNA level of MCT4. ∗*p* < 0.05, ∗∗*p* < 0.01 (n = 3) (**F**); ATP content in RAECs cytoplasm. ∗∗∗*p* < 0.001 (n = 3) (**G**); Mitochondrial respiration of RAECs was measured by Seahorse XFp. ∗∗*p* < 0.01 (n = 3) (**H**); Representative transmission electron microscopy (TEM) images of RAECs mitochondria, the arrows indicate mitochondria (**I**); Representative images of the mitochondrial membrane potential detected by JC-10, which was quantified by the red-green fluorescence ratio. ∗∗∗*p* < 0.001 (n = 7) (**J**).

In order to simulate the plateau environment, mice were placed for 45 days in a plateau simulated chamber with an oxygen concentration of about 9.0 % at an altitude of 6000 m. Endothelial function was evaluated by the vascular reactivity. Results showed the endothelium-dependent diastolic function of the thoracic aorta in mice was significantly impaired by the hypobaric hypoxic environment (Fig. 2A). Consistently, immunoblotting results revealed that the expression of eNOS and phosphorylated eNOS (p-eNOS), key enzymes for endothelial relaxation, in the thoracic aorta of hypobaric hypoxia mice was significantly decreased (Fig. 2B). We also observed decreased expression of proteins that maintain vascular endothelial barrier function, including claudin family proteins, tight junction protein ZO-1, and vascular endothelial VE-Cadherin (Fig. 2B). These results suggest that hypobaric hypoxia may impair vascular endothelial function.

To further characterize the influence of hypoxia on VECs, RAECs were cultured under 5 % O_2_. Consistent with in vivo studies, the expression of ZO-1, eNOS and p-eNOS also was significantly reduced in VECs exposed to hypoxia (Fig. 2C). Moreover, immunostaining demonstrated decreased eNOS under hypoxic exposure (Fig. 2D). In cellular energy metabolism, pyruvate is metabolized into lactate in cytoplasm and then excreted from cells via MCT4. Pyruvate also enters mitochondria through MPC1 to take part in the tricarboxylic acid cycle. To verify whether vascular endothelial dysfunction is associated with energy metabolism disruption, we performed lactate assay and Seahorse analysis. Hypoxia induced a marked increase in lactate level of cell culture medium supernatant (Fig. 2E), while it was observed that was a significant upregulation in MCT4 expression (Fig. 2F). The results of total intracellular ATP content detection showed that hypoxia caused a significant increase in ATP content (Fig. 2G). These results all suggest hypoxia may lead to increased glycolytic flux in VECs. In fact, in subsequent experiments, we also visually observed that hypoxia caused a significant rise in glycolytic flux by examining ECAR (Fig. S1). Additionally, hypoxia resulted in abnormal levels of oxidative phosphorylation and mitochondrial morphology, characterized by a significant decrease in mitochondrial ATP production (Fig. 2H), and partial disappearance of mitochondrial cristae and membrane rupture (Fig. 2I). At the same time, we used JC-10 staining for mitochondrial membrane potential to demonstrate a significant decrease in the mitochondrial red/green fluorescence ratio following RAECs hypoxia (Fig. 2J), indicating enhanced mitochondrial membrane depolarization. Therefore, we postulate that hypoxia disturbs the balance between vascular glycolysis and oxidative phosphorylation, subsequently contributing to mitochondrial and vascular endothelial dysfunction.

### 3.3 Blocking PDK improves vascular endothelial dysfunction through promoting oxidative phosphorylation

**Fig. 3:**
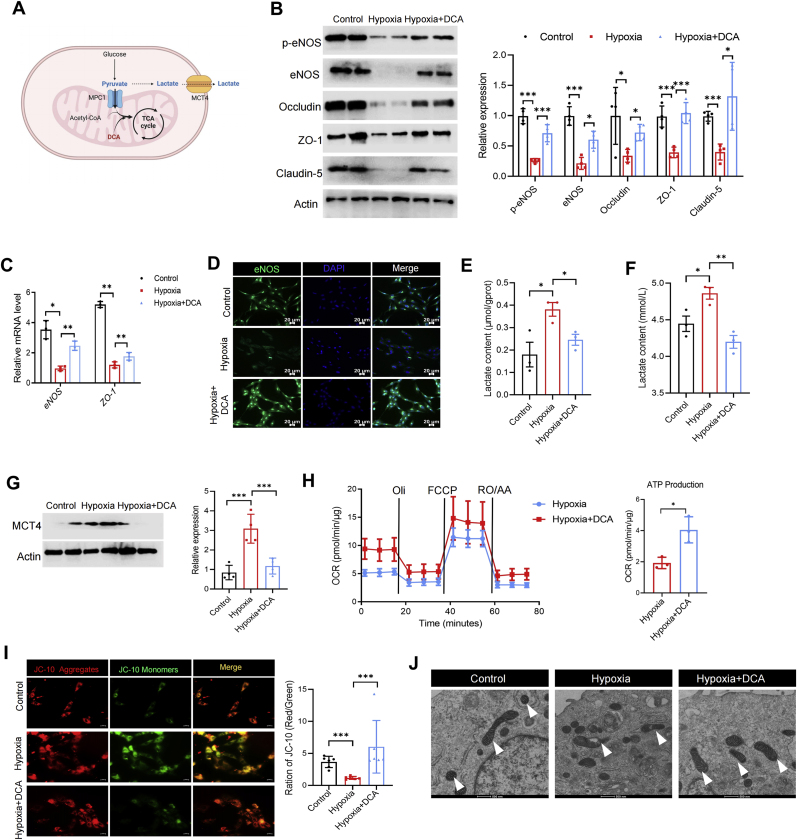
**Blocking PDK improves vascular endothelial dysfunction through promoting oxidative phosphorylation in hypoxia exposure VECs.** RAECs was treated with dichloroacetate (DCA, 3 mM) for 24 h and then cultured under hypoxic conditions for 72 h, DCA can force pyruvate to enter oxidative phosphorylation (**A**); The protein expression of p-eNOS, eNOS, Occludin, ZO-1 and Claudin-5 was analyzed. ∗*p* < 0.05, ∗∗∗*p* < 0.001 (n = 4) (**B**); The transcription level of eNOS and ZO-1. ∗*p* < 0.05, ∗∗*p* < 0.01 (n = 3) (**C**); Representative images of immunofluorescence analysis of eNOS expression (**D**); Lactate content in the cytosol of RAECs, normalized using intracellular protein content. ∗*p* < 0.05 (n = 3) (**E**); Lactate content in the supernatant of RAECs culture. ∗*p* < 0.05, ∗∗*p* < 0.01 (n = 3) (**F**); The protein expression of MCT4. ∗∗∗*p* < 0.001, (n = 4) (**G**); Relative mitochondrial respiration of RAECs under different treatments was measured. ∗*p* < 0.05 (n = 3) (**H**); Representative images of the mitochondrial membrane potential of RAECs detected by JC-10 after different treatments, mitochondrial membrane potential was quantified by red-green fluorescence ratio. ∗∗∗*p* < 0.001 (n = 6) (**I**). TEM images of RAECs mitochondria after different treatments, the arrows indicate mitochondria (**J**).

In VECs, glucose enters lactate metabolism pathway mainly through glycolysis. A small amount of pyruvate enters mitochondria to participate in oxidative phosphorylation. Within mitochondria, pyruvate is converted to acetyl-CoA by pyruvate dehydrogenase complex (PDHC). Because pyruvate dehydrogenase kinase (PDK) can inhibit the catalytic activity of PDHC on the oxidation of pyruvate, so blocking PDK can activate mitochondrial oxidative phosphorylation [14]. In order to investigate whether increased glycolytic flux and decreased oxidative phosphorylation flux caused by hypoxia promotes vascular endothelial dysfunction, we used DCA, a specific inhibitor of PDK, to inhibit the activity of PDK and force pyruvate to enter oxidative phosphorylation (Fig. 3A). We found that VECs treated with DCA exhibited higher levels of eNOS and p-eNOS, as well as increased expression of tight junction proteins such as Occludin, ZO-1, and Claudin-5 compared with hypoxic VECs (Fig. 3B). Furthermore, similar patterns of changes were also obtained by RT-qPCR (Fig. 3C). Immunofluorescence assay also confirmed that DCA significantly recovered expression of eNOS in RAECs exposed to hypoxia (Fig. 3D). Consistent with the changes in eNOS, we also observed that the inhibition of PDK alleviated the reduction of NO release in RAECs caused by hypoxia (Fig. S2A). These results clearly indicate that blockade of PDK can alleviate vascular endothelial dysfunction triggered by hypoxia.

Subsequently, to investigate whether DCA ameliorates energy metabolism disorders, we detected the lactate content in the cell culture medium. As expected, DCA treatment significantly reduced hypoxia-induced intracellular lactate elevation, while also significantly reduced lactate release from RAECs under hypoxic conditions (Fig. 3E and F). In comparison to gene expression, the hypoxia-induced upregulation of MCT4 was significantly inhibited by DCA (Fig. 3G), indicating that DCA can prevent hypoxia-induced glycolysis enhancement. Expectedly, cell oxygen consumption rate results revealed that DCA indeed promoted the oxidative phosphorylation of RAECs versus the hypoxic group (Fig. 3H). In contrast, ECAR results showed that DCA treatment significantly reduced the glycolytic flux of RAECs compared with the hypoxic group (Fig. S1). We also observed that DCA treatment significantly reduced intracellular ATP content (Fig. S4F). These results all suggest that DCA can alleviate the disturbance of energy metabolism caused by hypoxia. Meanwhile, improved mitochondrial membrane potential in RAECs was also observed in DCA post-treatment cell (Fig. 3I). Mitochondrial transmission electron microscopy (TEM) results further demonstrated that DCA treatment notably reduced mitochondrial damage induced by hypoxia (Fig. 3J). Considering that mitochondrial damage can lead to increased ROS, we examined ROS levels within RAECs and showed that hypoxia caused a significant increase in ROS, while treatment of cells with DCA reduced ROS levels within RAECs (Fig. S3A). In summary, these findings indicate that inhibiting PDK can improve energy metabolism disturbance, thereby preserving vascular endothelial dysfunction.

To further verify that the energy metabolism disorder caused by hypoxia is an important factor of vascular endothelial dysfunction, we blocked key nodes of glycolytic pathway using different pharmacological inhibitors to investigate VECs' function under hypoxia exposure. First, under hypoxia, we blocked the glucose-lactate pathway using Sodium oxamate (SO), an inhibitor of lactate dehydrogenase (LDH), or 2-DG, an inhibitor of hexokinase (Fig. S4A), and endothelial function was subsequently tested. In line with findings in DCA treated cells, SO or 2-DG partially prevented hypoxia-induced eNOS and p-eNOS decline (Fig. S4B). Additionally, this treatment effectively enhanced the expression of RAECs’ tight junction protein and cell adhesion protein (Fig. S4C). Notably, compared with the hypoxic group, 2-DG treatment also reduced the release of lactate from the RAECs (Fig. S4D). Furthermore, the expression level of lactate carrier MCT4 in SO or 2-DG treated cells was significantly decreased compared to hypoxic cells (Fig. S4E). Similarly, we also observed that 2-DG significantly reduced intracellular ATP content (Fig. S4F). In subsequent studies performed in HUVECs, similar to findings in RAECs, it was showed that both the promotion of oxidative phosphorylation by DCA and the inhibition of glycolysis by SO alleviated hypoxia-induced vascular endothelial dysfunction (Fig. S4G–I). Altogether, these data suggest that maintenance of the vascular endothelial functional and integrity may greatly depend on energy homeostasis under hypoxia.

### 3.4 Knocking down MCT4 improves hypoxia-mediated vascular endothelial dysfunction

**Fig. 4:**
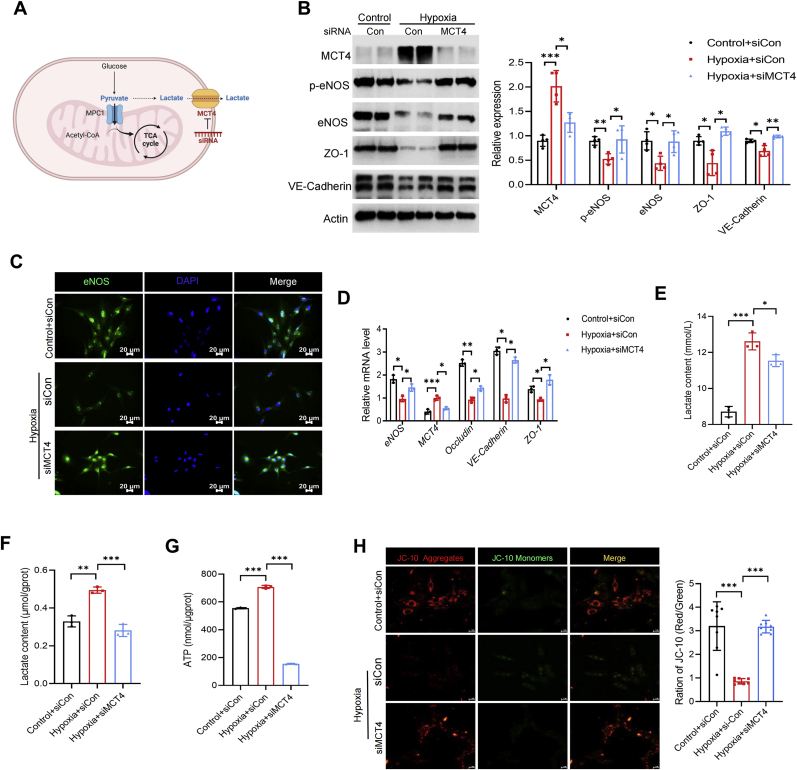
**Knocking down MCT4 improves hypoxia-mediated vascular endothelial dysfunction**. RAECs were transfected with MCT4 or control siRNA, then followed by culturing in 5 % O_2_ for 72 h. Diagram of cell energy metabolism after knocking down MCT4 using siRNA (**A**); The protein expression of MCT4, eNOS, p-eNOS, ZO-1 and VE-Cadherin in RAECs was detected. ∗*p* < 0.05, ∗∗*p* < 0.01, ∗∗∗*p* < 0.001 (n = 4) (**B**); Representative images of immunofluorescence analysis of eNOS (**C**); Relative transcription levels of eNOS, MCT4, Occludin, VE-Cadherin and ZO-1 in RAECs under different treatments. ∗*p* < 0.05, ∗∗*p* < 0.01, ∗∗∗*p* < 0.001 (n = 3) (**D**); Lactate content in the supernatant of RAECs culture. ∗*p* < 0.05, ∗∗∗*p* < 0.001 (n = 3) (**E**); Lactate content in the cytosol of RAECs, normalized using intracellular protein content. ∗∗*p* < 0.01, ∗∗∗*p* < 0.001 (n = 3) (**F**); ATP content in RAECs cytoplasm, normalized using intracellular protein content. ∗∗∗*p* < 0.001 (n = 3) (**G**); Representative images of the mitochondrial membrane potential of RAECs detected by JC-10 after different treatments, mitochondrial membrane potential was quantified by red-green fluorescence ratio. ∗∗∗*p* < 0.001 (n = 8) (**H**).

MCT4 plays an important role in lactate transport by controlling the release of cellular lactate. Additionally, it is a vital component of the pyruvate-lactate axis. To determine if this axis influences RAECs energy metabolism under hypoxia, we using siRNA to knock down MCT4 (Fig. 4A). Immunoblotting analysis revealed that, when compared with the hypoxic group, the levels of eNOS and p-eNOS associated with endothelium-dependent relaxation, significantly increased in RAECs following MCT4 depletion (Fig. 4B). Furthermore, proteins linked to endothelial permeability, like ZO-1 and VE-Cadherin, also were significant recovered (Fig. 4B). Immunofluorescence further revealed that the fluorescence intensity of eNOS was enhanced in RAECs with MCT4 knockdown (Fig. 4C). RT-qPCR analysis revealed that MCT4 knockdown mitigated the hypoxia-induced reduction in eNOS, ZO-1, Occludin, and VE-Cadherin mRNA levels (Fig. 4D). In addition, we also observed that knocking down MCT4 alleviated the reduction of NO release from RAECs caused by hypoxia (Fig. S2B). To investigate if MCT4 knockdown could ameliorate the energy metabolism disruption induced by hypoxia, we measured lactate levels in the RAECs culture medium. The data indicated that MCT4 knockdown resulted in a decrease in lactate levels both intracellularly and in the cell culture medium supernatant, compared with the hypoxic group (Fig. 4E and F). As expected, after MCT4 was knocked down, intracellular ATP levels also decreased significantly (Fig. 4G). Additionally, improved mitochondrial membrane potential was observed in post-MCT4 knockdown cell following hypoxia exposure (Fig. 4H). As expected, knockdown of MCT4 also alleviated the increase in ROS levels caused by hypoxia (Fig. S3B). These findings suggest that knocking down MCT4 can reduce energy metabolism disorders and vascular endothelial dysfunction caused by hypoxia.

### 3.5 Blocking MPC1 impairs vascular endothelial function by disrupting energy homeostasis

**Fig. 5:**
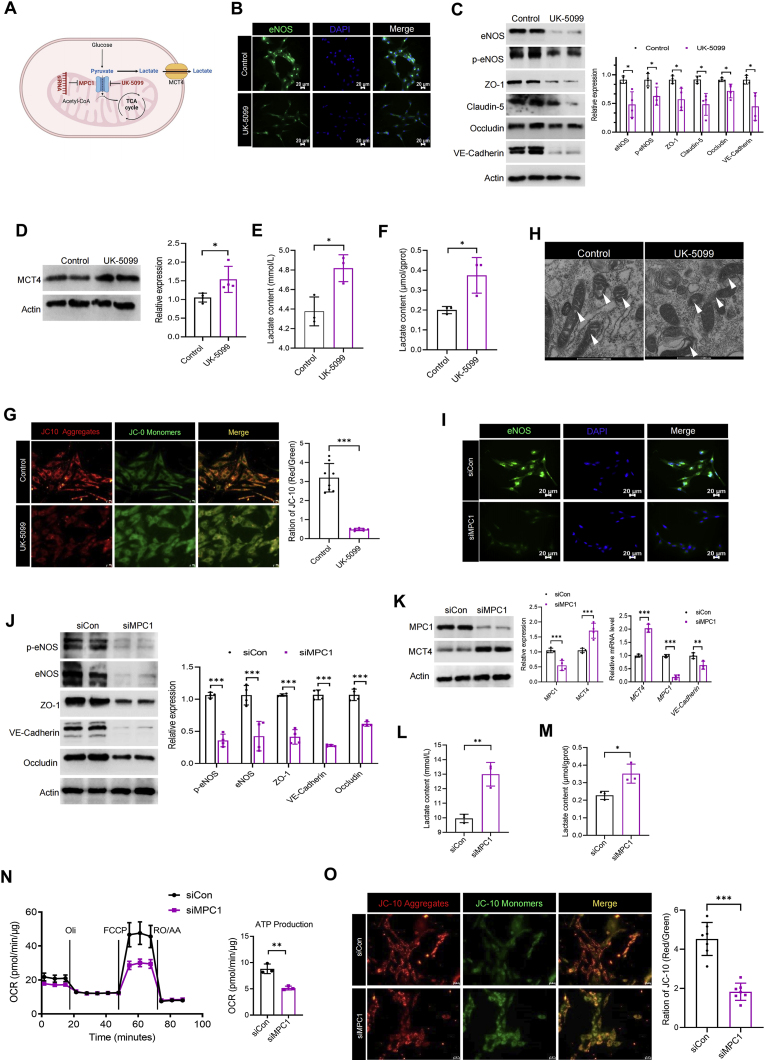
**Blocking MPC1 impairs vascular endothelial function though disrupting energy homeostasis.** Diagram of cell energy metabolism after blocking MPC1 using siRNA or UK-5099 (10 μM) (**A**); (**B–H**) RAECs was treated with UK-5099 for 24 h to block MPC1. Representative images of immunofluorescence analysis of eNOS (**B**); The protein expression of eNOS, p-eNOS, ZO-1, Claudin-5, Occludin and VE-Cadherin was detected. ∗*p* < 0.05 (n = 4) (**C**); The protein expression of MCT4 in RAECs. ∗*p* < 0.05 (n = 4) (**D**); Lactate content in the supernatant of RAECs culture. ∗*p* < 0.05 (n = 3) (**E**); Lactate content in the cytosol of RAECs, normalized using intracellular protein content. ∗*p* < 0.05 (n = 3) (**F**); Representative images of the mitochondrial membrane potential of RAECs detected by JC-10, mitochondrial membrane potential was quantified by red-green fluorescence ratio. ∗∗∗*p* < 0.001 (n = 8) (**G**); Representative TEM images of RAECs mitochondria, the arrows indicate mitochondria (**H**); (**I–O**) RAECs were transfected with MPC1 siRNA to knock down MPC1. Representative images of immunofluorescence analysis of eNOS in RAECs after knocking down MPC1 (**I**); The protein expression of eNOS, p-eNOS, ZO-1, Occludin and VE-Cadherin was detected. ∗∗∗*p* < 0.001 (n = 4) (**J**); The protein and mRNA levels of MCT4 and MPC1 were detected. ∗∗*p* < 0.01, ∗∗∗*p* < 0.001 (n = 4) (**K**); Lactate content in the supernatant of RAECs culture. ∗∗*p* < 0.01 (n = 3) (**L**); Lactate content in the cytosol of RAECs, normalized using intracellular protein content. ∗*p* < 0.05 (n = 3) (**M**); The oxygen consumption rate (OCR) of RAECs after MPC1 knockdown was analyzed by Seahorse XF analyzer, and the ATP production was normalized using intracellular protein content. ∗∗*p* < 0.01 (n = 3) (**N**). Representative images of the mitochondrial membrane potential of RAECs detected by JC-10 after knocking down MPC1, and mitochondrial membrane potential was quantified by red-green fluorescence ratio. ∗∗∗*p* < 0.001 (n = 7) (**O**).

Pyruvate primarily depends on MPC1 for its entry into the mitochondria. To further examine the effects of disrupting the pyruvate-lactate axis on vascular endothelial function, RAECs were treated with the MPC1-specific inhibitor UK-5099 for 24 h (Fig. 5A). Following this treatment, we observed a significant reduction in the expression levels of both eNOS and p-eNOS (Fig. 5B and C). We also observed that inhibition of MPC1 resulted in a reduction of NO content in RAECs (Fig. S2C). Additionally, adding UK-5099 in RAECs disrupted tight junctions, as demonstrated by a notable decrease in the expression of Occludin, ZO-1, Claudin-5, and VE-Cadherin (Fig. 5C). Interestingly, we discovered that after treating with UK-5099, MCT4 expression (Fig. 5D) and lactate content (Fig. 5E and F) also significantly increased, suggesting a correlation between MCT4 and MPC1. These findings imply MCT4 and MPC1 jointly regulating the homeostasis of the pyruvate-lactate axis in RAECs. Surprisingly, after treatment with UK-5099, the intracellular ATP content exhibited a time-dependent change. Specifically, ATP levels significantly rose following 12 h of exposure to UK-5099, but subsequently dropped markedly after 24 h (Fig. S5A). As the treatment time increased with UK-5099, the intracellular ATP content first increased and then decreased, which may be associated with a reduction in cellular activity due to prolonged inhibition of MPC1. Following JC-10 staining, we observed a significant decrease in mitochondrial membrane potential (Fig. 5G), as well as pathological structural damage to mitochondria (Fig. 5H). These results suggest that blocking MPC1 can inhibit energy metabolism of RAECs through the pyruvate-lactate metabolic axis, ultimately leading to vascular endothelial dysfunction.

To further verify the role of MPC1 in vascular endothelial dysfunction, siRNA was used to knock down MPC1. Results revealed that the expression of eNOS and p-eNOS significantly decreased following the MPC1 knockdown (Fig. 5I and J). Correspondingly, we also observed that knocking down MPC1 led to a decrease in the NO content in RAECs (Fig. S2D). Similarly, the expression levels of Occludin, ZO-1 and VE-Cadherin also decreased significantly (Fig. 5J). All of these suggest that MPC1 knockdown leads to a severe vascular endothelial dysfunction. Moreover, we discovered that the expression of MPC1 and MCT4 are negatively correlated at both protein and mRNA levels (Fig. 5K), which further demonstrates that the fate of pyruvate influences the vascular endothelial function. In subsequent experiments, we examined the energy metabolism of RAECs following MPC1 knockdown. We observed that MPC1 knockdown significantly increased intracellular lactate and lactate release (Fig. 5L and M). Similar to the effects observed with long-term treatment using UK-5099, we found no significant increase in intracellular ATP content after MPC1 knockdown (Fig. S5B). However, in order to ensure knockdown efficiency, we were unable to detect intracellular ATP content within 12 h of transfection. On the other hand, MPC1 knockdown reduced mitochondrial ATP production (Fig. 5N) and led to a breakdown in mitochondrial membrane potential (Fig. 5O). In addition, we also observed that blocking MPC1 would lead to a significant increase in ROS levels in RAECs, which might be related to mitochondrial damage (Fig. S3C and D). These results suggest that MPC1 knockdown induces vascular endothelial dysfunction by disrupting energy metabolism homeostasis.

### 3.6 Administration of a PDK inhibitor improves vascular endothelial function in hypobaric hypoxia environment, while an MPC1 inhibitor drives vascular endothelial dysfunction

**Fig. 6:**
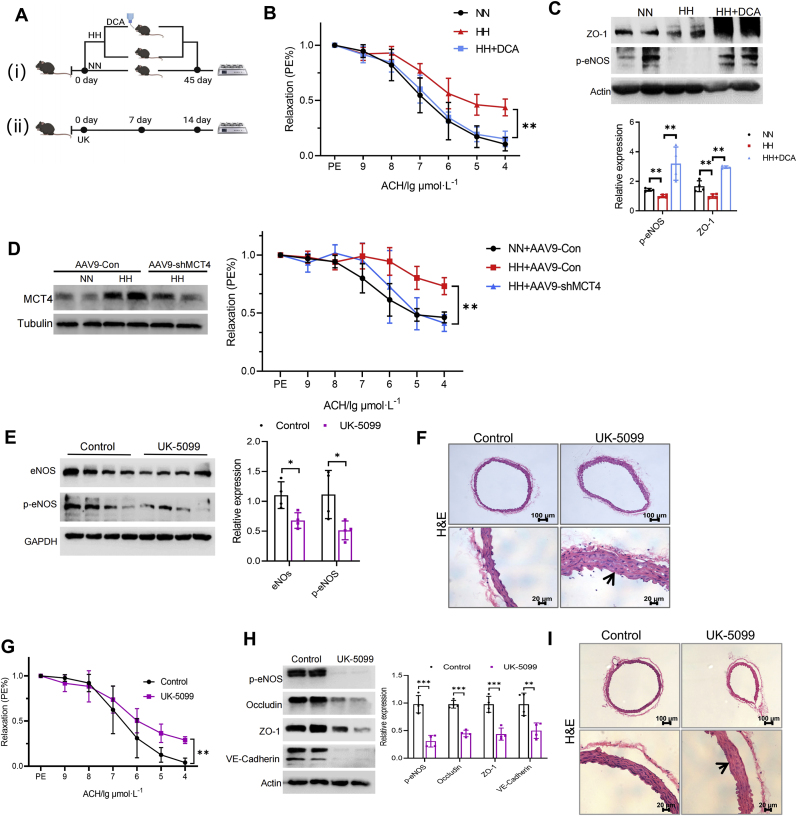
**Administration of a PDK inhibitor improves vascular endothelial function in hypobaric hypoxia environment, while an MPC inhibitor drives vascular endothelial dysfunction.** (**A**) Diagram of experiment. (i) Mice were randomly divided into three groups: Normoxia (NN), Hypobaric hypoxia (HH) and Hypobaric hypoxia + DCA (HH + DCA). After the mice adapted to the environment, the hypobaric hypoxia group and DCA group were put into a simulated chamber of hypobaric hypoxia. The normoxia group was fed at normal altitude. The mice in the DCA group were fed ddH_2_O containing DCA, while the other groups were fed normal diet. 45 days later, the thoracic aorta was collected for follow-up experiments. (ii) Mice were randomly divided into two groups: control and UK-5099. The administration group was injected intraperitoneally with UK-5099 every day for 7 and 14 days. "Endothelium-dependent relaxation in response to acetylcholine was measured in mice from NN, HH and HH + DCA. ∗∗*p* < 0.01 (n = 6) (**B**); The protein expression of p-eNOS and ZO-1 in thoracic aorta in mice from NN, HH and HH + DCA. ∗∗*p* < 0.01 (n = 4) (**C**); Mice were injected with AAV9-ENT-MCT4 shRNA or negative control intravenically, cultured under normal environment for 2 weeks, and then transferred to a hypobaric hypoxia simulated chamber for 45 days, and then the endothellar-dependent relaxation function of mice was measured. ∗∗*p* < 0.01 (n = 7) (**D**); The protein expression of p-eNOS and eNOS in thoracic aorta of control and UK-5099 treated mice for 7 days. ∗*p* < 0.05 (n = 4) (**E**); Representative images of pathological changes of thoracic aorta damages with H&E staining in mice treated with UK-5099 for 7 days (**F**); Endothelium-dependent relaxations to acetylcholine was measured in mice treated with UK-5099 for 14 days. ∗∗*p* < 0.01 (n = 6) (**G**); Relative expression of p-eNOS, Occludin, ZO-1 and VE-Cadherin in thoracic aorta of mice treated with UK-5099 for 14 days. ∗∗*p* < 0.01, ∗∗∗*p* < 0.001 (n = 4) (**H**); Representative images of pathological changes of thoracic aorta damages with H&E staining in mice treated with UK-5099 for 14 days (**I**).

To further characterize the crucial role of energy metabolism homeostasis dependent on the pyruvate-lactate axis in maintaining vascular endothelial function, we exposed mice to a simulated high-altitude environment of 6000 m and administered DCA to the experimental group, the experimental protocol is shown in Fig. 6A. After 45 days of exposure, the vasodilation function of the thoracic aorta of the mice was assessed. The results revealed that the vasodilation function of mice exposed to hypobaric hypoxia (HH) was impaired; however, the vasodilation function in administered DCA mice showed a significant recovery (Fig. 6B). At the same time, p-eNOS and barrier protein ZO-1 showed higher levels in the DCA administered mice compared to those in hypobaric hypoxia mice (Fig. 6C). Furthermore, MCT4 in VECs was knocked down using VECs-specific MCT4 shRNA adeno-associated virus (AAV-shMCT4) to infect mice, and AAV-shRNA Control (AAV-Con) was injected as control. After 2 weeks of virus injection, the mice were transferred to a hypobaric hypoxic simulated chamber for 45 days, and endothelium-dependent relaxation of the mice was subsequently detected. The results showed that, compared with the hypoxic group, MCT4 knockdown significantly improved the endothelium-dependent relaxation function of mice (Fig. 6D). These findings indicate that enhancing oxidative phosphorylation can protect vascular endothelial function during hypoxia exposure.

Subsequently, we administered UK-5099 in mice to disrupt pyruvate-lactate axis under normoxia (Fig. 6A). We then assessed the vasodilation function of their thoracic aortas on days 7 and 14. Interestingly, on day 7, while there were no significant changes observed in the endothelium-dependent vasodilation to ACH of the thoracic aorta, but both eNOS and p-eNOS exhibited the reduced expressions (Fig. 6E). Additionally, hematoxylin and eosin (H&E) staining revealed damage to the endovascular cortex (Fig. 6F). On the 14th day, the endothelium-dependent vasodilation to ACH of the thoracic aorta in UK-5099 administered mice notably declined (Fig. 6G), meanwhile, the expression levels of key proteins associated with vascular endothelial function including p-eNOS, Occludin, ZO-1, and VE-Cadherin also diminished significantly (Fig. 6H). The examination of H&E staining revealed that the endovascular cortical folds in the UK-5099 treated mice had partially disappeared and were disrupted (Fig. 6I). These results suggest that hypobaric hypoxia causes energy metabolism imbalance and vascular endothelial dysfunction by disrupting the homeostasis of pyruvate-lactate axis.

### 3.7 Energy metabolism disorders caused by hypoxia are mediated at least partly by PKM2

**Fig. 7:**
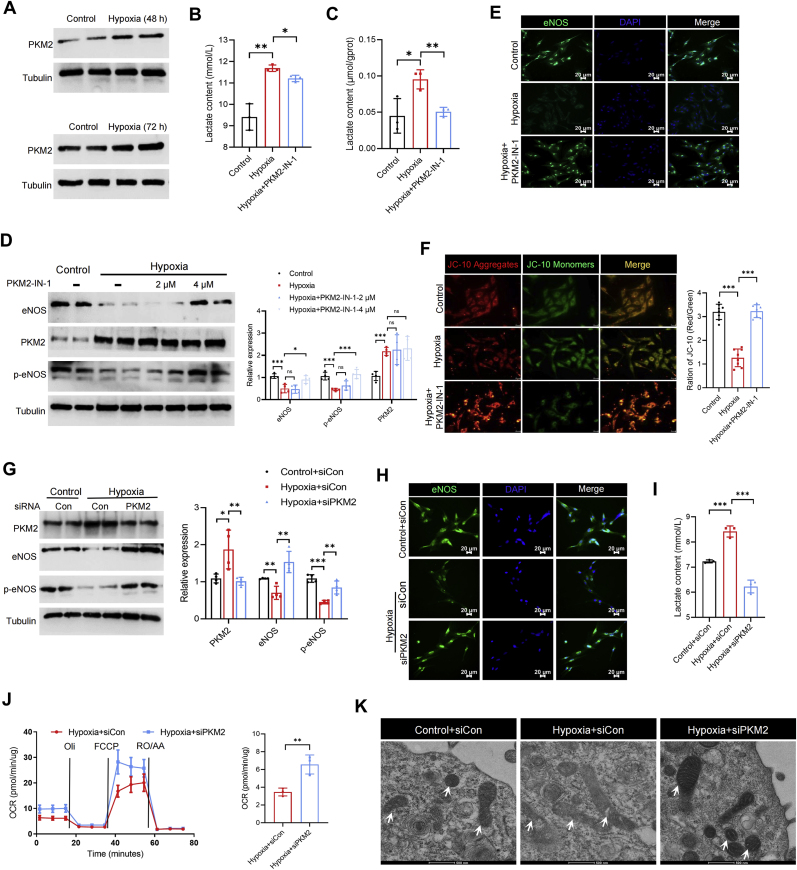
**Blocking PKM2 alleviates vascular endothelial dysfunction caused by hypoxia by inhibiting energy metabolism disorders.** Relative expression of PKM2 in RAECs cultured under hypoxic conditions for 48 h and 72 h (**A**); (**B–F**) RAECs was treated with PKM2-IN-1 at a final concentration of 2 and 4 μM for 24 h, respectively, and then cultured under 5 % O_2_ for 72 h. Lactate content in the supernatant of RAECs culture. ∗*p* < 0.05, ∗∗*p* < 0.01 (n = 3) (**B**); Lactate content in the cytosol of RAECs, normalized using intracellular protein content. ∗*p* < 0.05, ∗∗*p* < 0.01 (n = 3) (**C**); The protein expression of eNOS, PKM2 and p-eNOS in RAECs sunder different treatments. ∗*p* < 0.05, ∗∗∗*p* < 0.001 (n = 4) (**D**); Representative images of immunofluorescence analysis of eNOS in RAECs (**E**); Representative images of the mitochondrial membrane potential detected by JC-10, and mitochondrial membrane potential was quantified by red-green fluorescence ratio. ∗∗∗*p* < 0.001 (n = 8) (**F**). (**G-K**) RAECs were transfected with siRNA for 24 h and then cultured in 5 % O_2_ for 72 h. The protein expression of PKM2, eNOS and p-eNOS in RAECs under different treatments. ∗*p* < 0.05, ∗∗*p* < 0.01, ∗∗∗*p* < 0.001 (n = 4) (**G**); Representative images of immunofluorescence analysis of eNOS in RAECs (**H**); Lactate content in the supernatant of RAECs culture. ∗∗∗*p* < 0.001 (n = 3) (**I**); OCR of RAECs after PKM2 knockdown was analyzed by Seahorse XF analyzer, and the production of ATP was quantified. ∗∗*p* < 0.01 (n = 3) (**J**); Representative TEM images of RAECs mitochondria under different conditions, the arrows indicate mitochondria (**K**).

To further explore the mechanisms behind hypoxia-mediated vascular endothelial dysfunction, we examined key enzymes in the glycolytic pathway. Notably, the expression level of PKM2 was significantly increased after 48 h or 72 h of RAECs hypoxia, but other key enzymes slightly decreased under hypoxia (Fig. 7A; Fig. S6A), suggesting that PKM2 may play an important role in hypoxia-mediated energy metabolism disorders of VECs. We then treated RAECs for 24 h with PKM2-IN-1, a specific inhibitor of PKM2, and cultured RAECs under hypoxic condition for 72 h. We observed that inhibition of PKM2 also reduced hypoxia-mediated lactate elevation (Fig. 7B and C). Furthermore, we found that inhibiting PKM2 mitigated vascular endothelial dysfunction caused by hypoxia in RAECs (Fig. 7D and E; Fig. S2A). Interestingly, this inhibition also helped preserve the mitochondrial membrane potential in RAECs subjected to hypoxia (Fig. 7F), suggesting that PKM2 regulates not only glycolytic pathways but also oxidative phosphorylation in RAECs. Similarly, inhibiting PKM2 can also slightly alleviate the increase in ROS levels caused by hypoxia (Fig. S3A). To further explore PKM2's regulation to the entire energy metabolic pathway, we utilized siRNA for knocking down PKM2 under hypoxia. The results showed that PKM2 knockdown significantly up-regulated the expression of eNOS and p-eNOS (Fig. 7G and H) and increased intracellular NO production (Fig. S2B). Likewise, reducing PKM2 expression led to a notable decrease in lactate content (Fig. 7I). These results all suggest that PKM2 may promote glucose to lactate conversion under hypoxia, which appears to be in contrast to findings that PKM2 inhibits glycolytic flux [15]. To this end, we transfected plasmids to overexpress PKM2 (PKM2^OE^) in RAECs (Fig. S6B and C), and directly examined the effect of PKM2 accumulation on the glycolytic flux of RAECs. Interestingly, we found that at 24 h after transfection, glycolytic flux increased significantly, while at 36 h after transfection, consistent with previous studies, the glycolytic flux of RAECs no longer changed significantly, but its glycolytic energy decreased significantly (Fig. S6D and E). Subsequently, to explore whether PKM2 regulates oxidative phosphorylation, we measured the oxygen consumption rate in RAECs following PKM2 knockdown, and found that PKM2 knockdown markedly increased mitochondrial ATP under hypoxia (Fig. 7J). Finally, observing mitochondrial ultrastructure revealed that PKM2 knockdown significantly alleviates pathological phenomena like mitochondrial membrane dissolution and cristae deletion during hypoxia (Fig. 7K). Similarly, knocking down PKM2 can also reduce ROS levels of RAECs under hypoxic conditions (Fig. S3B). These results confirm that the elevated expression of PKM2 under hypoxia is a primary cause of vascular endothelial energy metabolism, eventually leading to vascular endothelial dysfunction.

### 3.8 PKM2 lactylation worsens energy metabolic imbalance leading to vascular endothelial dysfunction

**Fig. 8:**
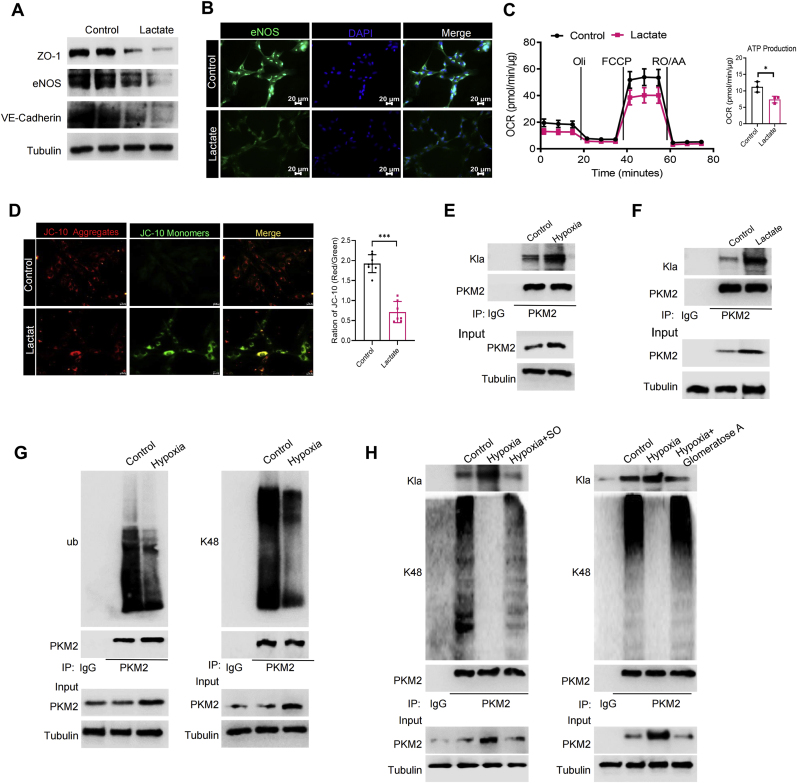
**PKM2 lactylation worsens energy metabolic imbalance leading to vascular endothelial dysfunction.** Relative protein expression of eNOS, ZO-1 and VE-Cadherin in RAECs treated with lactate (20 μM) (**A**); Representative images of immunofluorescence analysis of eNOS in RAECs treated with lactate (**B**); The OCR of RAECs treated with lactate was analyzed by Seahorse XF analyzer, and the production of ATP was quantified. ∗*p* < 0.05 (n = 3) (**C**); Representative images of the mitochondrial membrane potential of RAECs treated with lactate detected by JC-10, and mitochondrial membrane potential was quantified by red-green fluorescence ratio. ∗∗∗*p* < 0.001 (n = 8) (**D**); Changes in PKM2 lactylation after hypoxia exposure in RAECs (**E**); Changes in PKM2 lactylation after lactate stimulation in RAECs (**F**); Changes in PKM2 ubiquitination after hypoxia exposure in RAECs (**G**); Lysates were immunoprecipitated using anti-PKM2 and subsequently immunoblotted with antibodies against L-Lactyl Lysine (Kla) or K48 in RAECs treated with SO or Glomeratose An under hypoxia (**H**).

In all our results, we found that vascular endothelial dysfunction caused by hypoxia consistently accompanied elevated lactate levels. To determine whether lactate can trigger vascular endothelial dysfunction, RAECs were incubated with exogenously added lactate for 24 h. The results showed that adding 20 mM lactate to the culture medium affected protein expression related to endothelial function in RAECs. Specifically, there was a decrease in the levels of proteins such as ZO-1, eNOS, and VE-Cadherin (Fig. 8A and B) confirming lactate contributed to vascular endothelial dysfunction. As expected, lactate reduced the ATP production in RAECs mitochondria (Fig. 8C), and extracellular lactate accumulation also caused mitochondrial membrane potential damage (Fig. 8D).

Lactate can derive proteins lactylation by post-translationally modifying specific lysine residues, to alter the expression and function of proteins [16]. A recent study identified PKM2 as a lactylation substrate [17]. Therefore, based on our findings above in hypoxic RAECs and related researches, we examined whether hypoxia-producing lactate may affect PKM2 expression via its lactylation. Indeed, PKM2 lactylation significantly increased under RAECs exposed to hypoxia (Fig. 8E). Consistently, exogenous lactate also enhanced the PKM2 lactylation (Fig. 8F). Next, we explored whether the PKM2 lactylation was able to affect its protein level. We examined PKM2 ubiquitination following treating RAECs with proteasome inhibitor MG132, and found that PKM2 ubiquitination decreased significantly after hypoxia (Fig. 8G). By contrast, after inhibiting lactate production under hypoxia using LDH inhibitors SO or Glomeratose A, we discovered an inverse relationship between the PKM2 lactylation and ubiquitination level (Fig. 8H), suggesting the PKM2 lactylation prevents its ubiquitination degradation to enhance PKM2 expression. These results indicate that higher lactate content during hypoxia increases PKM2 lactylation to inhibit its ubiquitination degradation. Consequently, this further promotes glycolysis through a positive feedback loop, thereby exacerbating the energy metabolic imbalance and dysfunction in VECs.

## 4 Discussion

Acute exposure to plateau creates hypobaric hypoxia environment, which increases the risk of high-altitude illness (HAI) in under-acclimated individuals [18,19], the primary causative factor is hypoxia [[20], [21], [22]]. Hypoxia also seems to be a common feature of CVD, thus exacerbating adverse changes and promoting disease progression [23]. VECs play an important role in maintaining vascular homeostasis, and endothelial dysfunction is considered to be a feature of the initial stage of CVD [24]. In this study, we found that hypoxia caused disturbance of energy metabolism homeostasis. Further analysis reveals that hypoxia leads to a preference for glycolysis rather than oxidative phosphorylation of vascular endothelial energy metabolism, the outcome of these metabolic changes is the consequent lactate overproduction and vascular endothelial dysfunction. Excessive lactate enhances PKM2 lactylation, a key glycolytic enzyme, thereby reducing the ubiquitination and degradation of PKM2 in turn promoting glycolysis via a positive feedback loop, while mitochondrial function and oxidative phosphorylation further collapse, aggravating the vascular endothelial dysfunction. Blockage of the pyruvate-lactate axis can maintain the balance between glycolysis and oxidative phosphorylation to protect vascular endothelial function upon hypoxic condition. Therefore, we conclude that hypoxia mediates vascular endothelial dysfunction partly through pyruvate-lactate axis-dependent energy metabolism disturbances.

Regulating vascular tension is an important function of VECs. In healthy VECs, l-arginine produced relaxing factor NO through eNOS [25], which induces vasorelaxation by activating guanylate cyclase. In addition, VECs can also release vasoconstrictor factors, and the decline of vasodilator factors or the rise of vasoconstrictor factors released by VECs are signs of endothelial dysfunction and constitute the main cause of the development of many CVD [26]. In this study, we found that hypobaric hypoxia exposure leads to decreased vasodilation function of thoracic aorta in mice, and also observed decreased expression level of eNOS. VECs also act as a semi-permeable selection barrier between vascular smooth muscle and vascular lumen, ensuring material exchange between tissues and blood. The integrity of the endothelial barrier also plays an important role in maintaining vascular homeostasis. Loss of endothelial barrier integrity during various diseases (including sepsis, ischemia, and trauma) can lead to high vascular permeability and vascular swelling/edema, and various physiological and pathophysiological stimuli can also cause acute and chronic changes in endothelial permeability [26]. Consistently, we observed that hypoxia leads to decreased expression of endothelial barrier components such as ZO-1, Claudin-5, and VE-Cadherin, which means that hypoxia destructs vascular endothelial integrity and further exacerbates vascular endothelial dysfunction.

Although VECs are direct contact with blood, they require less oxygen than other tissues, and their energy source is mainly glycolysis driven by PFKFB3 [27], followed by oxidative phosphorylation involving fatty acid oxidation [28] and TCA cycle. It is precisely because of this special energy metabolism of VECs that the role of oxidative phosphorylation of VECs has been ignored for a long time. In fact, endothelial cell metabolism disorders can cause a series of cardiovascular diseases. For example, ablation of endothelial Sirtuin 3 changes glycolysis by disrupting glucose transport from VECs to cardiomyocytes, leading to pressure overload and heart failure [29]. Inhibition of glycolysis with 2-DG induces cytotoxicity of VECs [30]. Notably, our data support the idea that hypoxia might drive a shift in the way VECs metabolize energy. Studies of RNA-seq and targeted metabolomics have shown that hypoxia leads to enhanced glycolytic flux in VECs and decreased levels of oxidative phosphorylation, including the TCA cycle. Several lines of evidence support that HIFs are one of the reasons for the reduction of oxidative phosphorylation and the upregulation of glycolytic enzymes and glucose transport in hypoxic reactions [31]. Interestingly, we found a new mechanism for the switch of vascular endothelial energy metabolism pathway in hypoxic environment, that the change of pyruvate-lactate axis caused by hypoxia is the main cause of vascular endothelial energy metabolism disorder.

The pyruvate-lactate axis was first found to regulate cardiac hypertrophy and heart failure [32]. In cellular energy metabolism, pyruvate is produced by glycolysis, and part of pyruvate is converted to lactate by lactate dehydrogenase (LDH) and excreted by MCT [33], while the other part of pyruvate enters mitochondria by MPC [34] to participate in TCA cycle and oxidative phosphorylation. Previously, it has been shown that hypoxia can modulate the expression of MPC and MCT related subunits, for example, Wang et al. illustrated that hypoxia induced lactate secretion and glycolytic flux by down-regulating MPC levels [35], while the promoter of MCT4 can be activated by HIF-1α, which mediates the upregulation of MCT4 under hypoxia [36]. Similarly, we found that hypoxia-induced abnormal changes in the expression of key channel proteins MCT4 and MPC1 involved in the pyruvate-lactate axis. Thus, we suspect that hypoxia directly leads to the disturbance of the pyruvate-lactate axis. Indeed, blocking MCT4 could limit the release of lactate and rescue the vascular endothelial dysfunction caused by hypoxia, whereas direct blocking of MPC1 can induce vascular endothelial dysfunction. This suggests that the pyruvate-lactate axis is involved in maintaining vascular endothelial function. More interestingly, we also observed that hypoxia could result in severe mitochondrial damage, along with a significant decrease in ATP produced by mitochondrial oxidative phosphorylation. Oxidative stress caused by mitochondrial damage under hypoxia may also be one of the factors that aggravate endothelial damage [37]. In this respect, we revealed that blocking MCT4 under hypoxia can inhibit mitochondrial damage, while directly blocking MPC1 can also cause mitochondrial injury. Therefore, we suggest that mitochondrial dysfunction in VECs is associated with disturbances in the pyruvate-lactate axis. In general, hypoxia disrupts the pyruvate-lactate axis of VECs, leading to increased glycolysis and decreased levels of oxidative phosphorylation, thereby mediating vascular endothelial dysfunction.

Lactate is not only the end product of glycolysis, but also a signaling molecule. In VECs, lactate acts as a signaling molecule to activate NF-κB [36]. Consistently, the present study demonstrated hypoxia causes lactate overproduction, while lactate treatment can directly cause vascular endothelial dysfunction. In recent years, a new epigenetic modification, lactylation [16], has been discovered, further demonstrating the status of lactate's signaling molecules. The substrate of lactylation is not limited to histones. Studies have shown that lactylation of PKM2 increases its pyruvate kinase activity and reduces its tetramer to dimer transition and nuclear distribution [17]. In this study, we found that PKM2 is specifically elevated in VECs exposed to hypoxia, where it apparently contributes to elevated glycolysis, meanwhile lactate-mediated PKM2 lactylation inhibits PKM2 ubiquitination and degradation, thereby its pyruvate kinase further aggravating vascular endothelial energy metabolism disorder via a positive feedback loop. Inhibition of LDH by chemical inhibitors or reduction of lactate release can inhibit the PKM2 lactylation to promote ubiquitination degradation, thereby alleviating vascular endothelial dysfunction caused by hypoxia.

In general, the present studies demonstrate that the pyruvate-lactate axis mediates vascular endothelial dysfunction caused by hypoxia through regulating vascular endothelial cell energy metabolism. Specifically, hypoxia produces large amounts of lactate, PKM2 lactylation can inhibit its degradation in turn enhancing endothelial cell energy metabolism disorders and exacerbating vascular endothelial dysfunction. Importantly, the modulation of the pyruvate-lactate axis can maintain the balance between glycolysis and oxidative phosphorylation to protect vascular endothelial function. Our findings suggest significant clinical implications, offering new avenues for maintaining vascular system stability and preventing vascular diseases in both altitude-related and pathological hypoxia.

### 4.1 Limitations of study

Despite of our novel and significant findings, however, there are several limitations that should be noted. In light of the fact that high-altitude environments are complex, *in vivo* study upon hypobaric hypoxia can't perfectly mimic plateau environment. There is a crosstalk in the metabolism of the body upon hypoxia condition, the relevance of the pathophysiological of vascular endothelial and other metabolism homeostasis requires further investigation.

## CRediT authorship contribution statement

**Yuyu Zhang:** Writing – original draft, Methodology, Investigation, Formal analysis, Conceptualization. **Jinghuan Wang:** Methodology, Formal analysis, Data curation, Conceptualization. **Mengting He:** Formal analysis. **Jiayao Liu:** Writing – review & editing. **Jialin Zhao:** Formal analysis. **JinTao He:** Writing – review & editing. **Caiyun Wang:** Writing – review & editing. **Yuhui Li:** Writing – review & editing. **Chenxi Xiao:** Writing – review & editing. **Chunxiang Fan:** Funding acquisition. **Jun Chang:** Writing – review & editing. **Xinhua Liu:** Methodology, Investigation, Funding acquisition, Conceptualization.

## Ethics declarations

All animals and the experimental protocol conformed to the Animal Welfare Act Guide for Use and Care of Laboratory Animals, and were approved by Institutional Animal Care and Use Committee (IACUC), Fudan University, China.

## Funding

This work was supported by National Key R&D Program of China (2023YFA1801200), Pudong New Area Leading Talents of TCM (PWRI2023-08), and Construction of TCM discipline in Pudong New Area (YC-2023-0202, YC-2023-0607).

## Declaration of competing interest

The authors declare that they have no known competing financial interests or personal relationships that could have appeared to influence the work reported in this paper.

## Data Availability

The data that has been used is confidential.
