# Supplementary material for: Hypobaric hypoxia-driven energy metabolism disturbance facilitates vascular endothelial dysfunction
Source: Redox Biol. 2025 May 17;84:103675. doi: 10.1016/j.redox.2025.103675 (PMC12147897; doi:10.1016/j.redox.2025.103675)
Supplement: Multimedia component 1 [file mmc1.docx]

**Supplemental materials and methods**

**Antibodies**

All antibodies were shown in **Table S1**.

**Western blotting**

Total proteins were extracted from cells and tissues using RIPA lysates containing 1% protease phosphatase inhibitors. After centrifugation at 4°C and 12000 g for 10 min, the supernatant was collected, and the concentration of protein samples was detected and adjusted using the BCA protein concentration detection kit. Equal amounts of protein (30 μg) were separated by 8-12% SDS-PAGE and transferred to NC membranes. The membrane was blocked with 5% skim milk and incubated at 4°C overnight with primary antibody and for 1 h with secondary antibody. The protein-specific signal was detected with a Bio-Rad imager and the bands were quantified by density analysis (ImageJ software).

**Quantitative real‑time PCR**

RNA was isolated using TRIzol reagent (Takara) from RAECs and HUVECs. The concentration and purity of RNA was determined by Nanodrop, and 1000 ng RNA was reverse transcribed by PrimeScript™ Reverse Transcription Master mix (TaKaRa, RR036A). Real-time qPCR was performed using Hieff® qPCR SYBR Green Master Mix (Yeasen, 11201ES08, China). All of the reactions were performed at least in triplicate with β-actin or β-Tubulin as the control. Primers were shown in **Table S2**.

**Construction and infection of AAV-shRNA MCT4 (adeno-associated virus-endothelial specific system carrying shRNA against MCT4)**

The endothelial-specific promoter for intercellular adhesion molecule 2 (ICAM2) was cloned into the AAV vectors to construct ECs-specific MCT4 shRNA adeno-associated virus (AAV-shMCT4) (GeneChem Co., Ltd. Shanghai, China). 1 × 10^12^ vg AAV-shMCT4 was injected through tail-vein. Mice in the control group were injected with the corresponding volume of vaginal adenovirus. A follow-up experiment was performed 2 weeks after virus injection

**PKM2 overexpressing by plasmid in RAECs**

PKM2 overexpression plasmid (F: TGTAAAACGACGGCCAGT; R：CAGGAAACAGCTATGACC) was purchased from Miaolingbio (Wuhan, China). When the cell fusion degree was about 50%, the fresh medium was replaced and Lipofectamine 2000 (ThermoFisher Scientific, 11668-019) and PKM2 overexpression plasmid were added. Transfection efficiency was detected 24 h later and subsequent experiments were performed.

**Haematoxylin and eosin (H&E) staining**

The thoracic aorta of mice was isolated and the histopathological changes were observed. The separated thoracic aorta was cut into 2 mm segments and immediately fixed in 10% formalin solution for 24 h. The anatomical tissue is dehydrated and then fixed with paraffin wax. The sample is then cut to a thickness of 5 µm and stained with H&E dye.

**Immunofluorescence staining**

RAECs were pre-planted on slides, then the cell slides were fixed with 4% paraformaldehyde and then permeated with 0.25% Triton X-100 for 15 min. The cell slides were blocked with 10% goat serum for 0.5 h, then inverted and incubated them in a wet box for primary antibody and kept at 4°C overnight. After proper cleaning with PBS, the corresponding Fluor-conjugated secondary antibodies (ThermoFisher Scientific, A-11008) were added and incubated at room temperature for 1.5 h. The nucleus was stained with 4’6-diamino-2-phenylindole (DAPI). Images were captured using a fluorescence microscope (Carl Zeiss, Zeiss LSM780).

**Lactate level assay**

Lactate content in intracellular and cell medium supernatant was measured using Lactic Acid assay kit (Nanjing Jiancheng Bioengineering Institute, A019-2-1, China) according to the supplier's instructions. Briefly, the cell culture medium supernatant was centrifuged at 7500 r/min for 5 min, the sample was added to the reaction system quickly, and the termination solution was added after incubation at 37°C for 10 min. For the detection of lactate content in cells, cells were first collected and washed twice with pre-cooled PBS. Appropriate pre-cooled PBS was added, the cells were lysed using ultrasonic cell crusher (Scientz, SCIENTZ-IID), and then centrifuged at 7500 r/min for 5 min, and the supernatant was collected for detection. The absorbance was measured at 530 nm wavelength by enzymograph.

**Measurement of mitochondrial oxygen consumption (OCR) and extracellular acidification rate (ECAR)**

The mitochondrial oxygen consumption (OCR) and extracellular acidification rate (ECAR) of RAECs under different conditions was measured by Seahorse XFp analyzer (Agilent). Follow the manufacturer's instructions for using the Agilent Seahorse XFp Glycolysis Stress Test Kit (Agilent, 103017-100) and Seahorse XF Cell mito stress Test kit (Agilent, 103010-100). Briefly, cells were cultured uniformly on Seahorse XFp microplates (Agilent, 103025-100) at a concentration of 10000 cells/well. The cells were washed twice with test medium (Agilent, 103575-100) and incubated in a CO_2_-free incubator at 37°C for 45 min. The microplates were then loaded into the analyzer, and the probes (Agilent, 103022-100) were successively added the appropriate drug according to the kit instructions before ECAR or OCR measurement. After the experiment, Seahorse XFp software was used to analyze the experimental data and quantify Glycolysis flux or mitochondrial associated ATP production.

**Measurement of ATP content**

The intracellular ATP content was measured using an ATP assay kit (Beyotime, China) according to the manufacturer's instructions. In brief, cells were lysed using ATP lysate provided by the kit, followed by centrifugation at 12000 g for 10 min at 4°C, and the supernatant was used for subsequent detection. Then take 100 μL ATP test solution according to the instructions, add 10 μL sample to detect RLU value. ATP content was corrected by intracellular total protein concentration.

**Intracellular NO release**

The release of NO in cells was detected under a fluorescence microscope using the NO fluorescence probe (DF-FM DA, Beyotime, China). Briefly, RAECs were cultured in 12-well plates, and RAECs were subjected to subsequent treatment when the cell density was moderate. After washing the cells three times with PBS, incubate the cells with DF-FM DA solution (diluted at 1:1000) for 20 min. Subsequently, the DAF-FM-DA working solution was removed and the cells were washed 3 times with PBS before the slides were sealed with antifade mounting medium and observed under fluorescence microscope.

**Intracellular ROS determination**

The generation of intracellular ROS was observed by DHE (ROS fluorescent probe, MCE, PD-MY 003). Dissolve DHE with DMSO and store it at -20°C. RAECs were cultured on cell slides. After the cells grew to an appropriate density, different treatments were carried out on RAECs. When staining, the DHE stock solution was diluted to 5 μM with the culture medium, and the cells were incubated with the DHE working solution at 37°C for 20 min. After washing with PBS three times, the ROS fluorescence images were immediately observed under a fluorescence microscope.

**Transmission electron microscope (TEM)**

The cells were gently scraped off, washed twice with PBS pre-cooled at 4°C, and then centrifuged to collect the precipitation. The cell precipitate was suspended in the electron microscope fixative solution (Servicebio, G1102, China), preserved away from light, and the transmission electron microscope ultrathin section was prepared by Servicebio. The mitochondrial microstructure was observed by transmission electron microscopy.

**Co-immunoprecipitation (Co-IP)**

Cells were lysed by NP-40 lysis buffer (10% NP40, 150 mM NaCl, 1 mM EDTA, 50 mM Tris, pH=8.0) containing proteinase inhibitors and phosphatase inhibitor. After incubated on ice for 30 min, lysate was centrifuged at 12000 g for 10 min and supernatant was collected. Protein concentration was measured using Pierce Rapid Gold BCA Protein Assay Kit (ThermoFisher Scientific, A53225). Appropriate amount of antibody was added based on instruction. After protein-antibody mixture was incubated on ice for 2 h, 25 μL of pre-washed Protein A/G PLUS-Agarose (Santa Cruz Biotechnology, sc-2003) was added, and the whole mixture was incubated at 4°C for overnight. Then the mixture was centrifuged at 2500 g for 3 min to get agarose beads. Agarose beads were washed and mixed with same amount of 2x loading Sample Buffer, and boiled at 95°C for 5 min. Then samples were ready for western blotting analysis**.**

**Liquid chromatography (LC)-MS/MS**

Cell metabolites were analyzed by targeted metabolomics by APExBIO Technology LLC. Briefly, cells were washed with PBS precooled at 4°C, then scrolled off, centrifuged at 1000 r/min to form cell masses, and stored in liquid nitrogen. Before loading the machine, 200 μL water MP vortex was added to the samples respectively. Then 800 μL methanol/acetonitrile solution (1:1, v/v) and 10 μL (SUCCINIC ACID-D6 internal standard) were added to vortex for 60 s, ultrasounded at low temperature for 30 min, repeated twice, placed at -20°C for 1 h to precipitate protein, 14000 r/min, centrifugated at 4°C for 20 min. The supernatant was freeze-dried and the sample was stored at -80°C. The samples were separated by Agilent 1290 Infinity LC ultra-high performance liquid chromatography (Agilent). Mobile phase: Liquid A was 10 mM ammonium acetate aqueous solution, liquid B was acetonitrile. The samples were placed in an automatic injector at 4°C, the column temperature was 45°C, the flow rate was 300 μL/min, and the injection volume was 2 μL. The relevant liquid phase gradient is as follows: 0-18 min, the linear change of liquid B from 90% to 40%; In 18-18.1 min, liquid B changed linearly from 40% to 90%. In 18.1-23 min, liquid B was maintained at 90%. The 5500 QTRAP mass spectrometer (AB SCIEX) was used for mass spectrometry in negative ion mode. 5500 QTRAP ESI source conditions are as follows: source temperature 450°C, ion Source Gas1 (Gas1): 45, Ion Source Gas2 (Gas2): 45, Curtain gas (CUR): 30, ionSapary Voltage Floating (ISVF)-4500 V; The ion pair to be measured was detected by MRM mode.

**RNA-seq analysis**

The cells were washed twice with pre-cooled PBS, then gently scraped off, centrifuged at 1000 r/min and collected, placing them in liquid nitrogen for preservation. Cell samples were sequenced by APExBIO Technology LLC. In brief, RNA was first isolated from cells and subsequently determined for purity and concentration using a NanoDrop ND-1000 UV-Vis spectrophotometer (NanoDrop Technologies Inc.). RNA integrity was measured on a Bioanalyzer 2100 system using the RNA Nano 6000 Assay kit (Agilent) and only samples with RIN greater than 8 were used for sequencing. The cDNA library was prepared using Illumina TruSeq^®^RNA Sample Preparation v2 kit (Illumina). After the library was qualified, Illumina HiSeq PE150 sequencing was performed according to the effective concentration of the library and data production requirements. The raw data obtained from the downstream machine were filtered for low quality. Filtered data were compare with the reference genome sequence to get the comparison reference genome by HISAT2. Transcript reconstruction was assembled using Stringtie software to obtain accurate transcript results and counted the expression of each gene or transcript. Then the count value of gene expression was normalized to TPM (Transcripts Per Kilobase of exon model per Million mapped reads) value.

**Detection of differentially expressed genes and functional enrichment analysis**

The differentially expressed genes were detected by using DESeq2 R package with the fold change >1.5 and adjusted p-value < 0.05 as cut-off. Then functional enrichment analysis of DEGs was performed by clusterprofiler R package.

**Analysis of metabolites and metabolic pathways**

The distribution of metabolites was analyzed using box diagram. Arrange a set of data from largest to smallest, and calculate the maximum, upper quartile Q3, median, lower quartile Q1, and minimum values respectively to reflect the degree of variation of the data within the group. In the box plot, the horizontal coordinate is the group name, and the vertical coordinate is the content information. T-test or ANOVA is used to calculate the difference between groups, and p-value is used to indicate its significance. The screened metabolites were uploaded to MetaboAnalyst for metabolic pathway enrichment Analysis via the pathway Analysis (MetPA) module. A comprehensive metabolic network was established through KEGG database, and the metabolic pathways were visualized by comparing the detected metabolites.

**
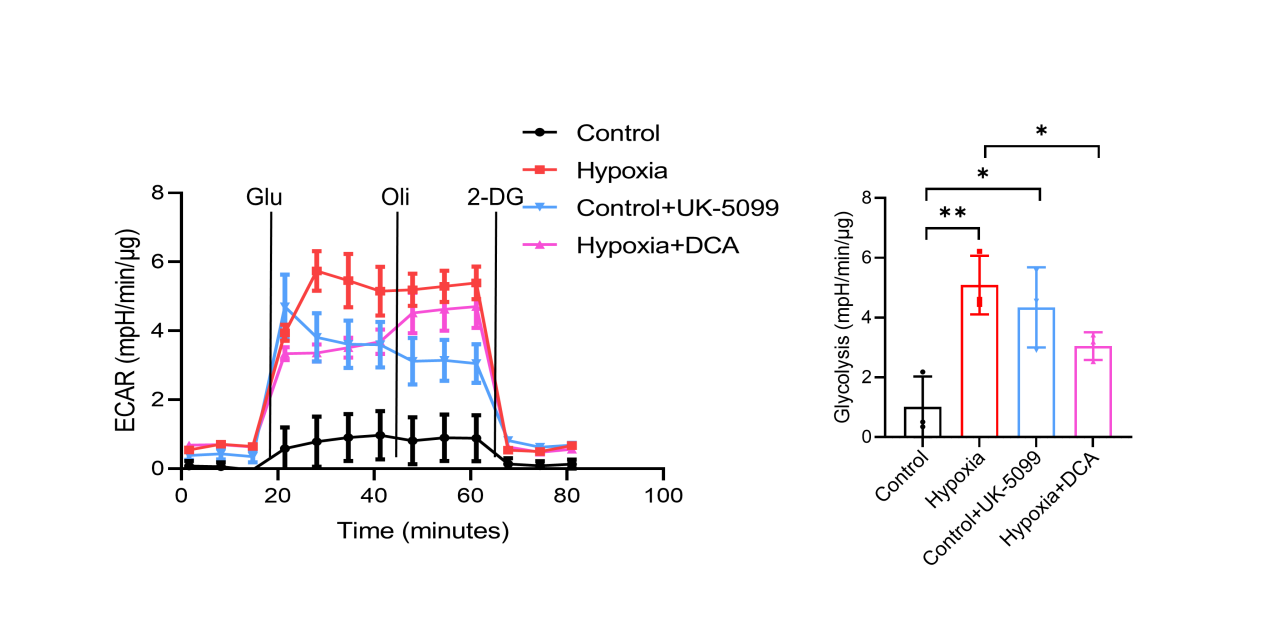
**

**Figure S1. The ECAR of RAECs under different treatments.** RAECs were treated with DCA (3 mM) or UK-5099 for 24 h, and cells were exposed to hypoxia for 72 h after DCA treatment. The extracellular acidification rate (ECAR) of RAECs was then detected, and normalized by the intracellular protein content. ^*^*p* < 0.05, ^**^*p* < 0.01 (n=3).

**Figure S2. The levels of NO in RAECs.**
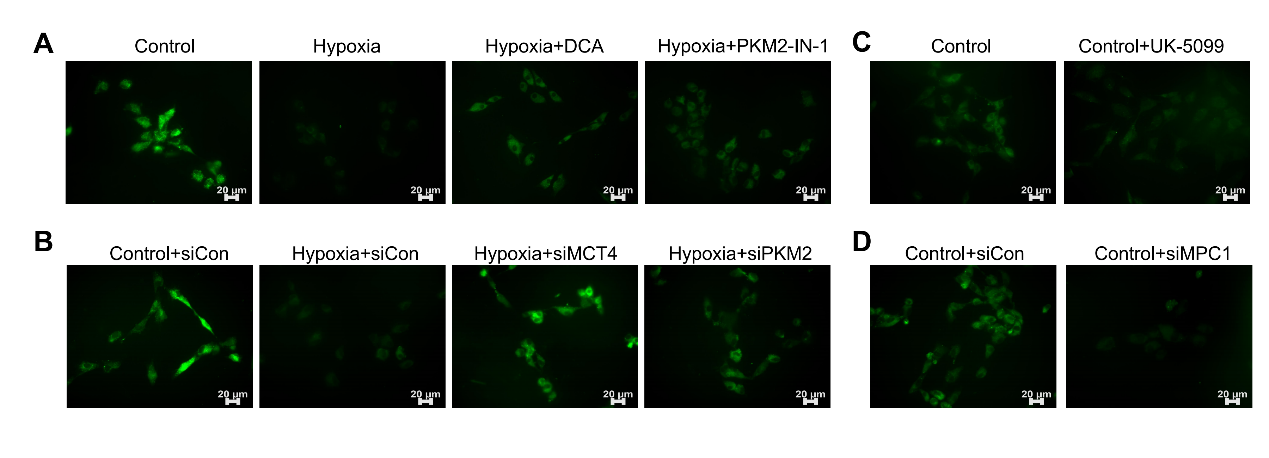
 RAECs were treated with DCA or PKM2-IN-1 and cultured in 5% O_2_ for 72 h, followed by DAF-FM DA staining (**A**); After knockdown of MCT4 and PKM2 with siRNA respectively, RAECs were cultured under hypoxia for 72 h, and then DAF-FM DA staining was performed (**B**); RAECs were treated with UK-5099 for 24 h and stained with DAF-FM DA (**C**); After knocking down MPC1 with siRNA, DAF-FM DA staining was performed on RAECs (**D**).

**Figure S3. The levels of ROS in RAECs**
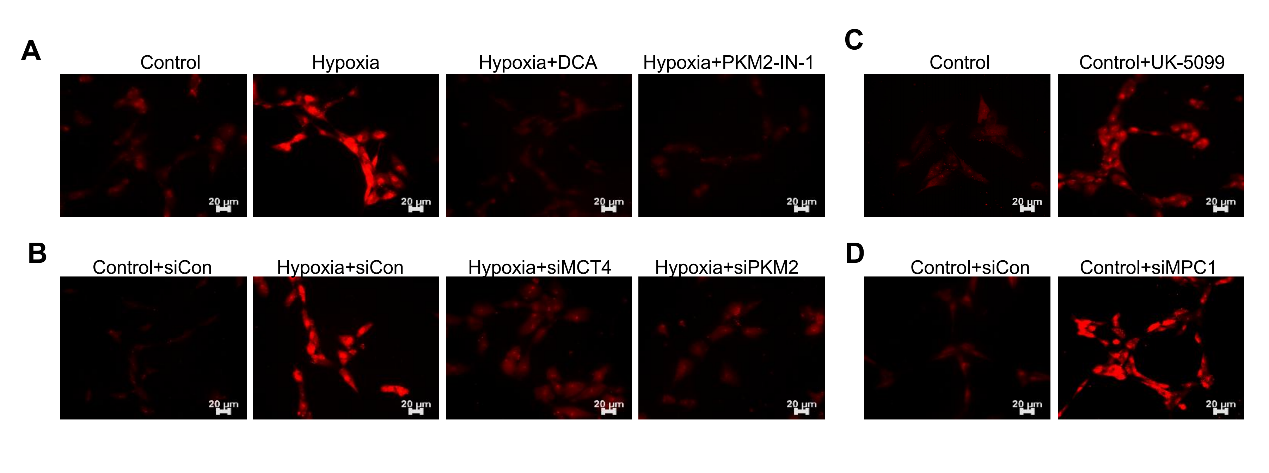
. RAECs were treated with DCA or PKM2-IN-1 and cultured in 5% O_2_ for 72 h, followed by DHE staining (**A**); After knockdown of MCT4 and PKM2 with siRNA respectively, RAECs were cultured under hypoxia for 72 h, and then DHE staining was performed (**B**); RAECs were treated with UK-5099 for 24 h and stained with DHE (**C**); After knocking down MPC1 with siRNA, DHE staining was performed on RAECs (**D**).

**
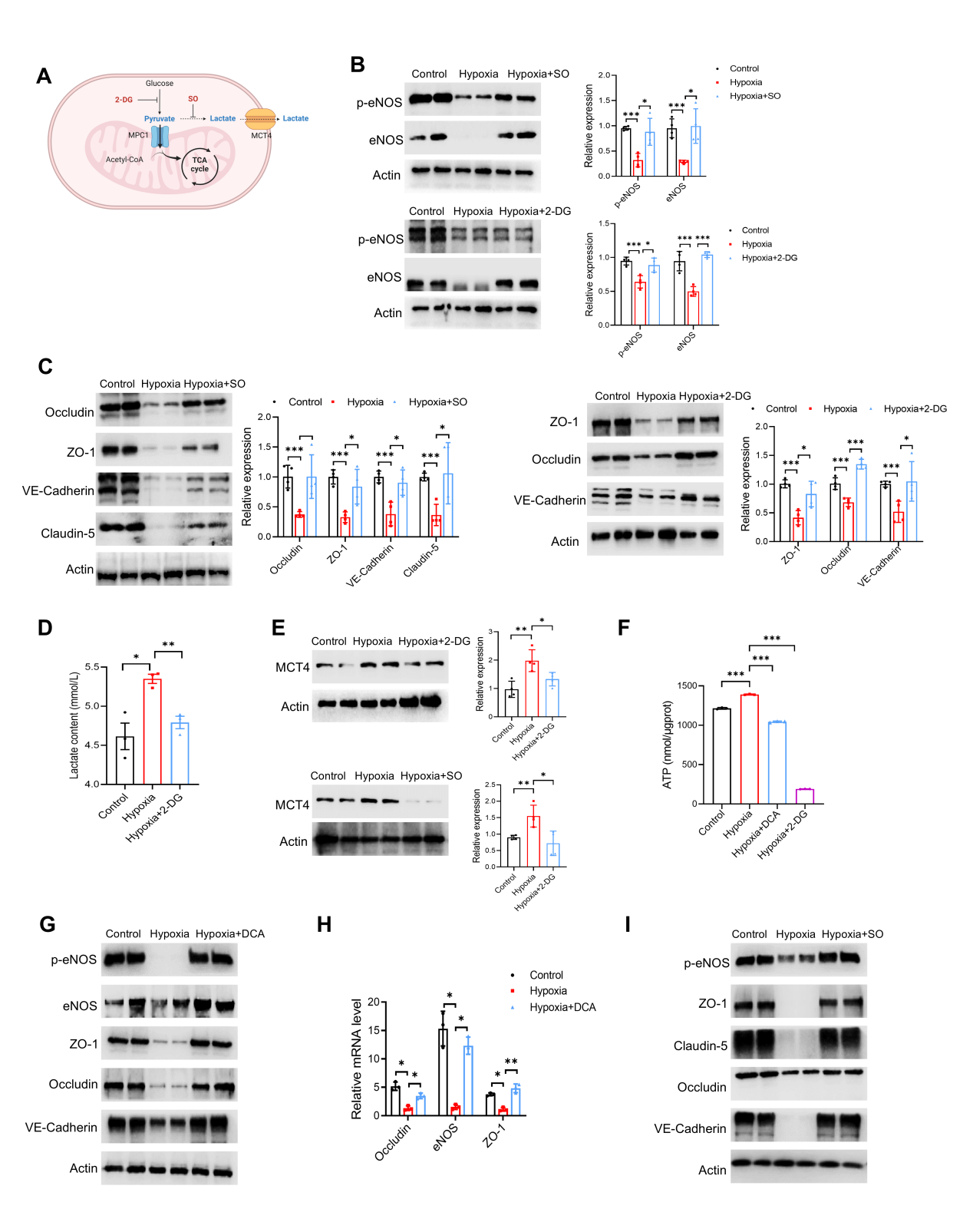
**

**Figure S4. Inhibition of glucose-lactate metabolic pathway alleviates endothelial dysfunction caused by hypoxia.** Diagram of cell energy metabolism after 2-DG (2 mM) and SO (50 μM) treatment (**A**). (**B-F**) RAECs were treated with 2-DG or SO for 24 h and then cultured under hypoxia for 72 h. Relative protein expression of eNOS and p-eNOS was detected. ^*^*p* < 0.05, ^***^*p* < 0.001 (n=4) (**B**); Relative protein expression of Occludin, ZO-1, Claudin-5 and VE-Cadherin was detected. ^*^*p* < 0.05, ^***^*p* < 0.001 (n=4) (**C**); Lactate content in the supernatant of RAECs culture under different treatments. ^*^*p* < 0.05, ^**^*p* < 0.01 (n=3) (**D**); Relative protein expression of MCT4 in RAECs was detected. ^*^*p* < 0.05, ^**^*p* < 0.01 (n=4) (**E**); ATP content in RAECs cytoplasm was normalized using intracellular protein content. ^***^*p* < 0.001 (n=3) (**F**). (**G-H**) HUVECs was treated with DCA (3 mM) for 24 h and then cultured at 5% O_2_ for 72 h, relative protein expression of p-eNOS, eNOS, Occludin, ZO-1 and VE-Cadherin was detected (**G**); mRNA level of eNOS, ZO-1 and Occludin was detected. ^*^*p* < 0.05, ^**^*p* < 0.01 (n=3) (**H**); HUVECs was treated with SO (50 μM) for 24 h and then cultured at 5% O_2_ for 72 h, relative protein expression of p-eNOS, eNOS, Occludin, ZO-1 and VE-Cadherin was detected (**I**).

**
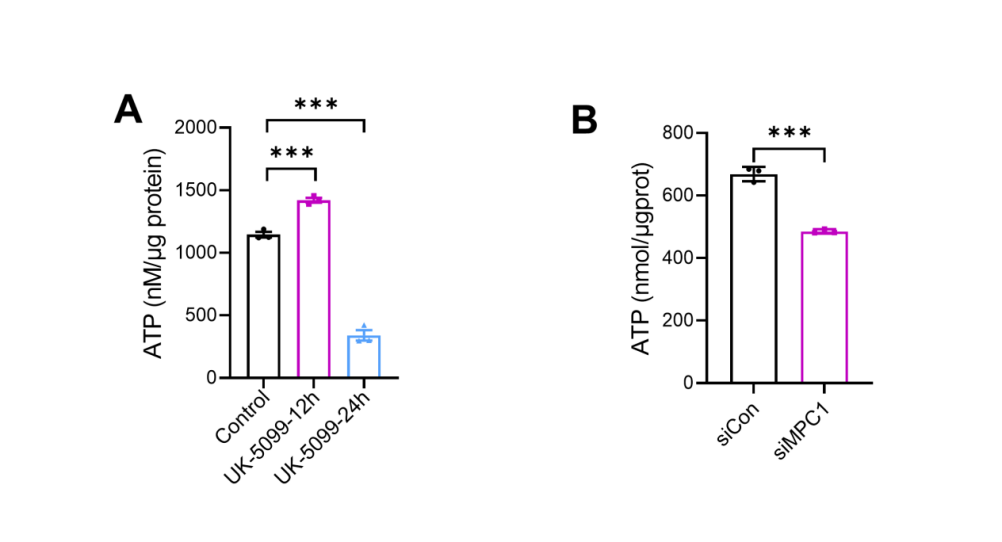
**

**Figure S5. ATP content in RAECs after blocking MPC1.** The ATP content in the cells was measured after 12 h and 24 h treatment of RAECs with UK-5099 and normalized by the protein content in the cells. ^***^*p* < 0.001 (n=3) (**A**); RAECs were transfected with MPC1 siRNA for 24 h, the intracellular ATP content was detected and the intracellular protein content was normalized. ^***^*p* < 0.001 (n=3) (**B**).

**
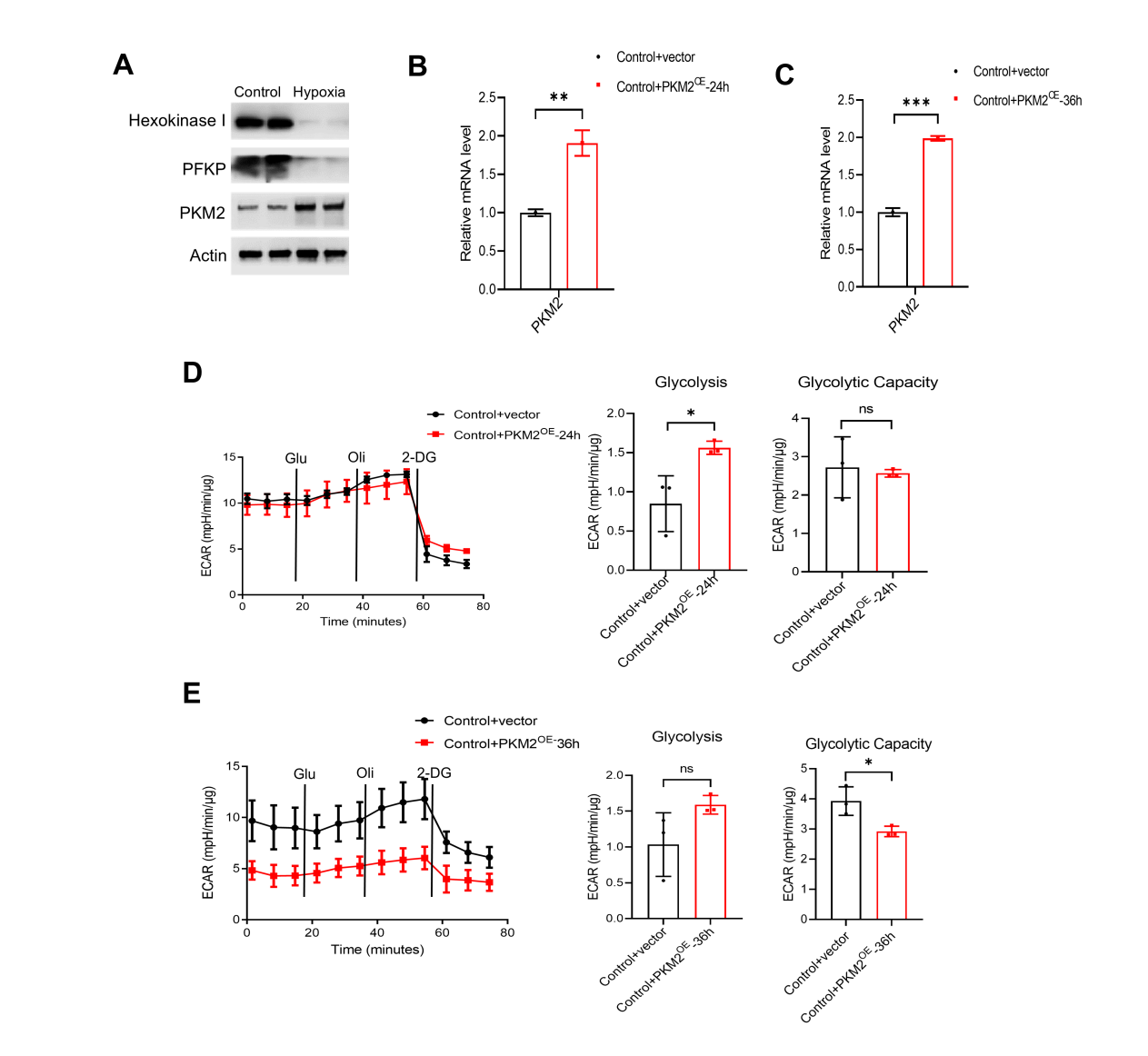
Figure S6.** RAECs were cultured under hypoxia for 72 h, relative protein expression of Hexokinase I, PFKP, PKM2 was detected (**A**); (**B-E**) Plasmid efficiency was verified and ECAR was examined after transfection of RAECs with plasmid for 24 h or 36 h, respectively. Relative levels of PKM2 in RAECs 24 h after transfection. ^**^*p* < 0.01 (n=3) (**B**); Relative levels of PKM2 in RAECs 36 h after transfection. ^***^*p* < 0.001 (n=3) (**C**); ECAR was detected 24 h after RAECs transfection and normalized by intracellular protein content. ^*^*p* < 0.05 (n=3) (**D**); ECAR was detected 36 h after RAECs transfection and normalized by intracellular protein content. ^*^*p* < 0.05 (n=3) (**E**).

**Table S1. The primary antibodies were used**

| Gene | Source | Item No. |
| --- | --- | --- |
| eNOS | ABclonal | A20985 |
| p-eNOS | Millipore | 07-428-I |
| ZO-1 | Proteintech | 21773-1-AP |
| VE-Cadherin | Invitrogen | 36-1900 |
| Claudin-5 | Abcam | ab131259 |
| Occludin | Proteintech | 27260-1-AP |
| MCT4 | Proteintech | 22787-1-AP |
| MPC1 | Cell Signal Technology | 14462S |
| PKM2 | Cell Signal Technology | 4053S |
| Hexokinase I | Cell Signal Technology | 2024T |
| PFKP | Cell Signal Technology | 8164T |
| β-Tubulin | Proteintech | 66240-1-lg, 10094-1-AP |
| β-Actin | Proteintech | 66009-1-Ig |
| GAPDH | Proteintech | 60004-1-Ig |
| Ub | Abways | CY5520 |
| K48 | Abways | CY5964 |
| L-Lactyl Lysine | Ptmab | PTM-1401RM |

**Table S2 Primers were used.**

| Primer name | Primer sequence (5´ to 3´) |
| --- | --- |
| Rat-β-actin-F | CATTGTCACCAACTGGGACGATA |
| Rat-β-actin-R | GGATGGCTACGTACATGGCTG |
| Rat _occludin_F | GAGAGATGCACGTTCGACCA |
| Rat _occludin_R | TCCTCCAAAGATGCCCGTTC |
| Rat _eNOS_F | AATTCTGGCAACAGAGGGCA |
| Rat _eNOS_R | AGGTGTTTCTTGGGTAGGCG |
| Rat _ZO-1_F | AGAAACCTCAAGCGAAGCCA |
| Rat _ZO-1_R | TCAGTTTCGGGTTTCCCCTT |
| Rat _VE-cadherin_F | CCAGAATTTGCCCAGCCCTA |
| Rat _VE-cadherin_R | GTCCTCGTTCTTCAGGGCAA |
| Rat _MPC1_F | GCCCTCTGTTGCTATTCTCTGAC |
| Rat _MPC1_R | GCCGCTTACTCATCTCGTAGTTG |
| Rat _MCT4_F | TCTTCCGAGACCGTGGCTTCC |
| Rat _MCT4_R | GGCACACCCATATCCTTAGCATAGC |
| Human_occludin_F | CTGCAAAGGGAAGAGCAGGA |
| Human_occludin_R | CCGCCAGTTGTGTAGTCTGT |
| Human_eNOS_F | GAAGCGAGTGAAGGCGACAA |
| Human _eNOS_R | AACTCTTGTGCTGTTCCGGC |
| Human_ZO-1_F | AGCCATTCCCGAAGGAGTTG |
| Human _ZO-1_R | GCAAAAGACCAACCGTCAGG |
| Human-GAPDH-F | TTCCACCCATGGCAAATTCC |
| Human-GAPDH-R | AGCATCGCCCCACTTGATTT |
